# The homeoprotein Dlx5 drives murine T-cell lymphomagenesis by directly transactivating Notch and upregulating Akt signaling

**DOI:** 10.18632/oncotarget.14784

**Published:** 2017-01-21

**Authors:** Yinfei Tan, Eleonora Sementino, Jinfei Xu, Jianming Pei, Zemin Liu, Timothy K Ito, Kathy Q Cai, Suraj Peri, Andres J.P. Klein-Szanto, David L Wiest, Joseph R Testa

**Affiliations:** ^1^ Cancer Biology Program, Fox Chase Cancer Center, Philadelphia, PA 19111, USA; ^2^ Histopathology Facility, Fox Chase Cancer Center, Philadelphia, PA 19111, USA; ^3^ Department of Biostatistics and Bioinformatics, Fox Chase Cancer Center, Philadelphia, PA 19111, USA; ^4^ Blood Cell Development and Function Program, Fox Chase Cancer Center, Philadelphia, PA 19111, USA

**Keywords:** T cell lymphoma, Notch, Akt, Dlx5, homeobox

## Abstract

Homeobox genes play a critical role in embryonic development, but they have also been implicated in cancer through mechanisms that are largely unknown. While not expressed during normal T-cell development, homeobox transcription factor genes can be reactivated via recurrent chromosomal rearrangements in human T-cell acute leukemia/lymphoma (T-ALL), a malignancy often associated with activated Notch and Akt signaling. To address how epigenetic reprogramming via an activated homeobox gene might contribute to T-lymphomagenesis, we investigated a transgenic mouse model with thymocyte-specific overexpression of the *Dlx5* homeobox gene. We demonstrate for the first time that *Dlx5* induces T-cell lymphomas with high penetrance. Integrated ChIP-seq and mRNA microarray analyses identified *Notch1/3* and *Irs2* as direct transcriptional targets of Dlx5, a gene signature unique to lymphomas from *Lck-Dlx5* mice as compared to T-cell lymphomas from *Lck-MyrAkt2* mice, which were previously reported by our group. Moreover, promoter/enhancer studies confirmed that Dlx5 directly transactivates Notch expression. *Notch1/3* expression and Irs2-induced Akt signaling were upregulated throughout early stages of T-cell development, which promoted cell survival during β-selection of T lymphocytes. Dlx5 was required for tumor maintenance via its activation of Notch and Akt, as tumor cells were highly sensitive to Notch and Akt inhibitors. Together, these findings provide unbiased genetic and mechanistic evidence that *Dlx5* acts as an oncogene when aberrantly expressed in T cells, and that it is a novel discovery that Notch is a direct target of Dlx5. These experimental findings provide mechanistic insights about how reactivation of the *Dlx5* gene can drive T-ALL by aberrant epigenetic reprogramming of the T-cell genome.

## INTRODUCTION

Aberrant expression of multiple NK-like (NKL) homeobox transcription factor genes is especially common in human T-cell acute leukemia/lymphoma (T-ALL), a malignancy often associated with activated Notch and Akt signaling [1]. While not expressed during normal T-cell development, NKL genes such as *TLX1* (*HOX11*), *TLX2* (*HOX11L1*), and *TLX3* (*HOX11L2*) may become transcriptionally activated in T-ALL [1]. Many of these developmentally related genes have also been implicated oncogenically, due to their reactivation via recurrent clonal chromosomal translocations and inversions. Typically, these rearrangements juxtapose T cell receptor regulatory sequences adjacent to homeobox loci such as *HOX11* [2] and *HOXA10* [3] leading to their upregulation. To date, however, little is known about oncogenic mechanisms and direct targets of these homeobox transcription factors in T-ALL.

The DLX family of homeodomain proteins also belong to the NKL superfamily. DLX homeoproteins play a role in bone formation, neurogenesis and hematopoiesis [4]. DLX5 was first identified as the mediator of bone morphogenetic protein (BMP) signaling and shown to regulate osteoblast differentiation, and *Dlx5* knockout mice exhibited defects in facial-cranial development [5]. Recently, DLX family members have been implicated in oncogenesis. For example, DLX5 is abundantly expressed in a subset of adult human T-cell lymphomas [6], and DLX5 may contribute to tumorigenesis by directly regulating *MYC* expression [7]. The role of DLX homeoproteins has also been extended to other malignancies. In lung cancer, upregulated expression of DLX5 is predictive of a poor prognosis, and knockdown of *DLX5* suppresses lung tumor cell proliferation [8]. In breast cancer, *DLX* homeoproteins have been shown to enhance metastatic potential, and DLX4 is capable of regulating epithelial-to-mesenchymal transition by augmenting TWIST levels [9]. Similarly, in glioblastoma patients, upregulation of DLX2 promotes tumor cell proliferation and is associated with reduced patient survival [10]. In ovarian cancer, DLX5 promotes cell proliferation via upregulation of AKT signaling through the direct transactivation of insulin receptor substrate 2 (*IRS2*) [11].

In mice, Dlx5 is normally expressed in adult bone marrow and fetal liver hematopoietic cells, but its expression is absent in Thy1-positive thymocytes [12]. *Lck-MyrAkt2* transgenic mice expressing a constitutively active (myristylated) form of the Akt2 kinase specifically in immature T cells develop a high incidence of thymic T-cell lymphomas. These tumors frequently harbor a somatic, clonal inversion of chromosome 6 that results in the juxtaposition of enhancer elements in the T-cell receptor (TCR) β-chain gene, *Trb*, and *Dlx5* [6]. This rearrangement in *Lck-MyrAkt2* mice results in high levels of expression of Dlx5 in a tissue where it is not normally expressed. This reactivation of Dlx5 was proposed to facilitate tumor development by interfering with T-cell differentiation and providing a second “hit” critical in the malignant transformation of thymocytes.

To address whether Dlx5 itself could represent a direct driving force in T-ALL, and how epigenetic reprogramming via a homeobox gene might contribute to T-lymphomagenesis generally, we generated a transgenic mouse model with thymocyte-specific overexpression of *Dlx5*, and we found that these *Lck*-*Dlx5* mice develop thymic lymphomas with high penetrance. The tumors that arise have constitutive activation of Akt in association with loss of Pten, and are highly sensitive to combinatory inhibition of Myc and Akt signaling [13]. We now report that Notch1/3 expression and Akt signaling are activated throughout T cell development in *Lck-Dlx5* mice, and that tumor formation is associated with further intensification of Notch and Akt signaling. While *Notch1* is regarded as the master oncogene in T-ALL [14], an *in vivo* mechanism responsible for its aberrant upregulation has not been previously reported. Using an unbiased, integrated genomic approach, we demonstrate for the first time that *Notch1*, *Notch3*, and *Irs2* are direct transcriptional targets of Dlx5 in thymic T cells. Collectively, the experimental findings presented here provide mechanistic insights about how the reactivation of *Dlx5* gene can drive T-ALL through aberrant epigenetic reprogramming.

## RESULTS

### *Lck-Dlx5* transgenic mice develop disseminated T-cell lymphomas

**Figure 1:**
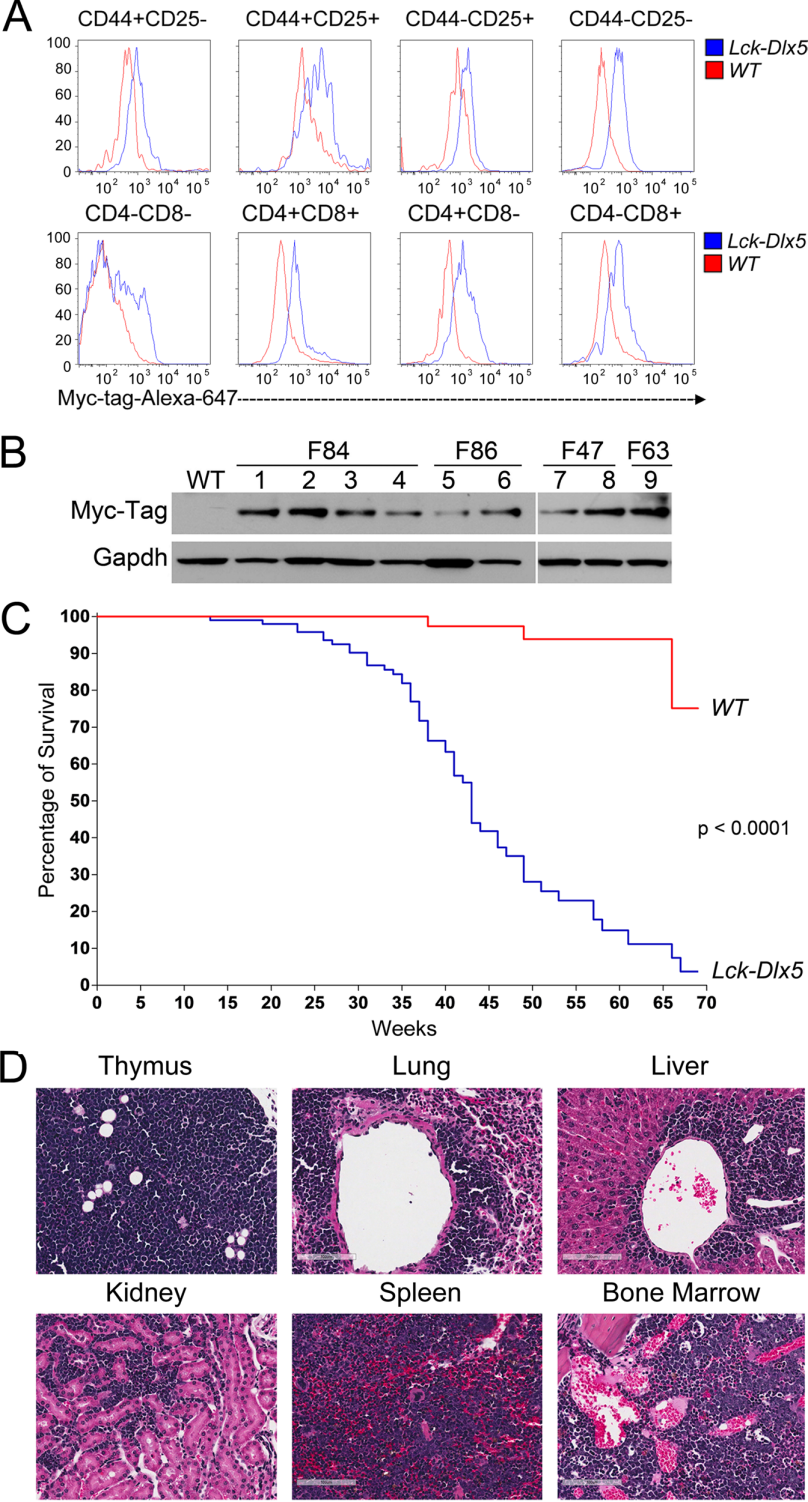
*Lck-Dlx5* mice express Dlx5 throughout T-cell development and are predisposed to disseminated T-cell lymphomas (**A**) representative flow cytometric analysis showing that Lck-driven, Myc-tag-labeled Dlx5 protein is expressed throughout thymic T-cell development. (**B**) expression of Myc-tagged Dlx5 in thymic lymphomas from four transgenic founder lines (F84, F86 [mouse# 801,793], F47 [mouse# 0, 918], F63 [mouse# 0]). (**C**) survival curve of *Lck-Dlx5* transgenic mice from founder line F86. (**D**) H&E staining showing representative T-cell lymphoma in thymus, lung, liver, kidney, spleen and marrow of *Lck-Dlx5* mice. Bars = 100 μm.

*Lck-Dlx5* transgenic mice were generated by injecting the *Lck-Myc-Tag-Dlx5* DNA fragment into blastocysts. Flow cytometric analysis revealed that non-malignant thymic T cells from all developmental stages expressed Myc-Tag Dlx5 protein (Figure 1A; Supplementary Figure 1A). *Lck-Dlx5* mice from each of four founders developed thymic lymphomas with high penetrance, and all tumors retained expression of Myc-tag Dlx5 (Figure 1B). Median survival of *Lck-Dlx5* mice founder line F86 was 41 weeks, F63 was 37 weeks and F84 was 32 weeks (Figure 1C, Supplementary Figure 1B). Primary tumors were diagnosed as thymic T-cell lymphomas based on H&E staining. The tumors often showed dissemination to the lung, with spreading to liver, kidney, spleen, marrow, and other organs in some animals (Figure 1D). Invasive tumor cells were mostly localized around blood vessels in lung, liver and kidney. IHC confirmed that primary and disseminated tumor cells were of T-cell origin, based on positive staining for CD3 (Supplementary Figure 1C), and that malignant cells were immature T cells, as they were CD4+CD8+ double positive (DP), similar to lymphoma cells in Lck-MyrAkt2 mice (Supplementary Figure 1D).

### Lymphomas in *Lck-Dlx5* mice exhibit a unique signature characterized by alterations of Notch and Akt

**Figure 2:**
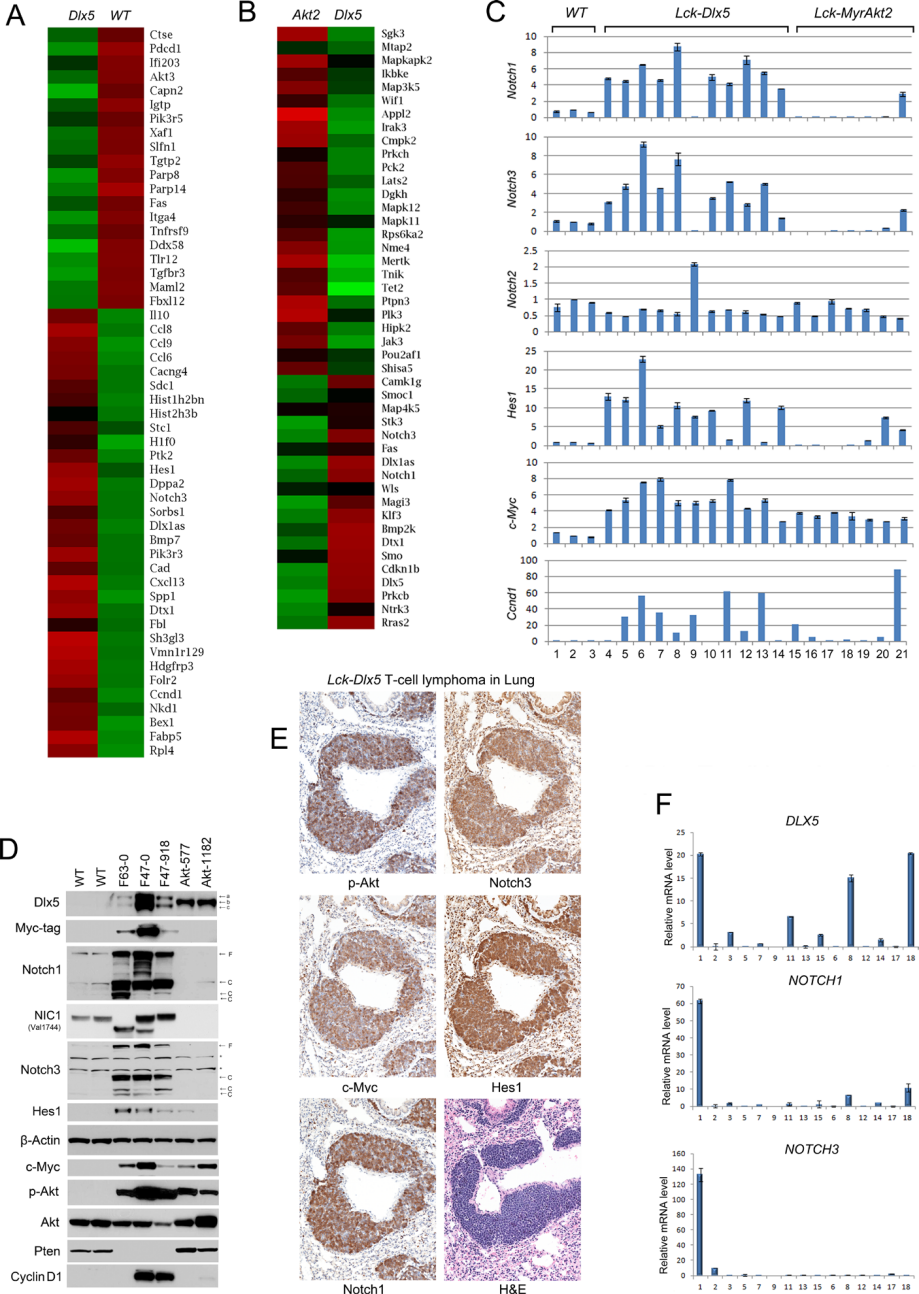
*Lck-Dlx5* lymphomas exhibit a molecular signature reminiscent of human T-ALL (**A**) RNA microarray heatmap demonstrating that compared to thymocytes from three WT mice, primary T-cell lymphomas from three *Lck-Dlx5* mice exhibit upregulation (*Red*) of genes involved in Notch signaling, cell cycle progression, chromatin modification, calcium homeostasis, glucose, fatty acid and nucleotide metabolism, as well as classical Dlx5 target genes. Down-regulated genes (*Green*) are implicated in pro-apoptotic events (*Tnf*, *Fas*, *Xaf1*, INFb signaling-related genes), tumor suppression, and DNA damage repair. (**B**) compared to T-cell lymphomas from *Lck-MyrAkt2* mice, *Lck-Dlx5* lymphomas show upregulation of *Notch1/3* and downregulation of *Lats2*, *Appl2*, *Jak3*, *Tet2*, and *Tnik*. Note that in the interests of journal space, the heatmap shown here includes only one sample from each group. The full heatmaps for all three samples examined per group can be found in Supplementary Figure 2C. (**C**) real-time PCR on three T-cell samples from WT mice (samples 1–3), 11 primary lymphomas from *Lck-Dlx5* mice (samples 4-14: F86-785, -801, -793, -1149; F63-0, -1263, -1210; F47-0, -1247, -918; and F84-1063), and 7 lymphomas from *Lck-MyrAkt2* mice (samples 15-21: F72-918, -2811, -3148, -3154; and F420-577, -1174, -1073), confirming unique upregulation of Notch signaling in Dlx5-driven tumors. Data = mean ± SEM. (**D**) immunoblot showing overexpression of Notch1, NIC1, Notch3, Hes1 and Myc in lymphoma lines (F63-0, F47-0, F47-918) from *Lck-Dlx5* mice. Loss of Pten expression and corresponding high levels of phospho-Akt in *Lck-Dlx5*-derived tumor cells are also shown, as are cyclin D1 levels. Dlx5 proteins have high molecular weight (a, b) and low molecular weight (c) forms. Notch proteins have full length (**F**) and cleaved (C) forms. *, non-specific bands. (**E**) H&E and immunohistochemical staining of lymphoma invading lung of *Lck-Dlx5* mouse. Note strong staining for Notch1, Notch3, Hes1, Myc and phospho-Akt in lymphoma. (F) summary of real-time PCR analysis of RNA from 15 pediatric T-ALL specimens showing expression levels of *DLX5*, *NOTCH1* and *NOTCH3*.

Karyotypic analysis revealed that trisomy 15 was the only recurring abnormality in *Lck-Dlx5* lymphomas (Supplementary Table 1; Supplementary Figure 2A). Array-CGH analysis uncovered unique rearrangements of *Tcrb* and *Tcra* loci in each *Lck-Dlx5* lymphoma examined, indicative of monoclonal proliferation of a malignant T cell in each tumor (Supplementary Figure 2B). We used Affymetrix microarrays to assess mRNA expression patterns in *Lck-Dlx5* lymphomas. These studies revealed recurrent upregulation of *Notch1, Notch3, Hes-1*, and *Ccnd1* in tumors from *Lck-Dlx5* mice, as compared to normal thymic T cells from WT mice (Figure 2A). To further discern factors potentially unique to Dlx5-driven lymphomagenesis, we compared gene expression profiles in lymphomas from *Lck-Dlx5* mice with those of *Lck-MyrAkt2* animals. This comparison revealed enhanced expression of Notch and histone cluster genes specifically in *Lck-Dlx5* lymphomas (Figure 2B). Sanger sequencing revealed that ~60% of tumors in *Lck-Dlx5* mice exhibited activating mutations in HD and PEST domains of *Notch1* (Supplementary Table 2). The commonly expressed genes in tumors from *Lck-Dlx5* mice and *Lck-MyrAkt2* mice, as well as uniquely expressed genes in those from *Lck-Dlx5* animals, were further categorized. Notably, expression of Ptk2/Fak was upregulated in both *Lck-Dlx5-* and *Lck-MyrAkt2-*derived lymphomas (Supplementary Figure 2C). In contrast, aberrant expression of Dlx1as, and Notch were a specific feature of *Lck-Dlx5* lymphomas, suggesting that a Dlx5-Notch-Myc axis plays a critical role in the pathogenesis of these tumors (Supplementary Figure 2D). Real-time RT-PCR confirmed that *Notch1*, *Notch3*, *Hes1* and *Myc* mRNA levels were upregulated in *Lck-Dlx5* lymphomas, when compared with those of WT thymic T-cells and *Lck-MyrAkt2* lymphomas (Figure 2C). Interestingly, the only *Lck-Dlx5* tumor (1/11) without overexpression of Notch1 and Notch3 instead showed upregulation of Notch2, further suggesting an essential role for aberrant Notch signaling in Dlx5-driven T-cell lymphomagenesis. *Ccnd1/Cyclin D1* mRNA expression was also frequently elevated in *Lck-Dlx5* tumors (Figure 2C). Moreover, immunoblotting demonstrated consistent upregulation of Notch1, Notch3, Myc, Hes1 and activation of Akt, as well as loss of Pten expression (Figure 2D; Supplementary Figure 2E). NIC1 specific antibody (Val1744) also revealed high levels of NIC1 in lymphoma cells from *Lck-Dlx5* mice, but no expression in tumors cells from *Lck-MyrAkt2* mice. The truncated NIC1 proteins seen in cell lines F63-0 and F86-793 (Figure 2D) correspond with our Sanger sequencing data, which revealed nonsense mutations that were predicted to result in protein truncations (Supplementary Table 2). Notably, these features resemble the major molecular signature of human T-ALL. Immunohistochemistry also confirmed that Notch1, Notch3, Hes1, Myc and phospho (active)-Akt are upregulated in primary and metastatic lymphomas from *Lck-Dlx5* mice (Figure 2E; Supplementary Figure 2F). Like human primary T-ALL, in which *TP53* mutations are rare [15, 16], lymphomas from *Lck-Dlx5* mice did not exhibit mutations in *Tp53* (not shown).

To determine if the findings in our murine model have clinical relevance, we performed real-time PCR on 15 pediatric T-ALL specimens and found that 3 samples had high levels of expression of *DLX5*. Notably, these three samples also had elevated expression of *NOTCH1*, and in one case, *NOTCH3* (Figure 2F). A search for published studies in expression repositories such as GEO and ArrayExpress websites revealed five studies of human T-cell ALL or T-cell lymphoma containing *DLX5* expression data. We found that DLX5 was over-expressed (z-score > 1) in 7–17% of cases in these studies, the exception being a smaller series by Iqbal et al. in which 2 of 5 cases expressed *DLX5*. Approximately 4% of samples showed higher expression of DLX5 based on Z-score > 2 [3, 17–19].

### Dlx5 is required for tumor maintenance via activation of Notch and Akt

**Figure 3:**
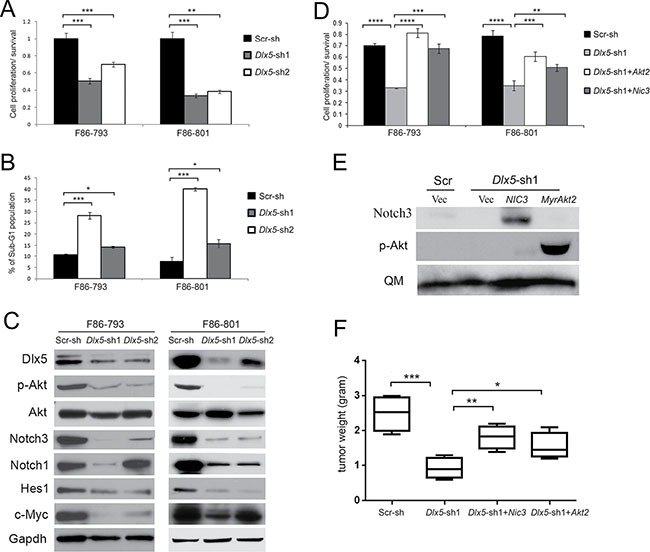
Dlx5 is required for survival of *Lck-Dlx5* lymphoma cells (**A**) lymphoma cells from *Lck-Dlx5* mice were transduced with two different retrovirus-mediated shRNAs against *Dlx5*, or with scrambled control shRNA (Scr). Tumor cell viability was reduced after *Dlx5* knockdown as shown by MTS assay. (**B**) Flow cytometry analysis depicting increased sub-G1 apoptotic population in lymphoma cells transduced with *Dlx5* shRNA. (**C**) immunoblotting revealing diminished full-length Notch1, Notch3, Myc, Hes1, and phospho-Akt levels following *Dlx5* knockdown. (**D**) MTS assay demonstrating rescue of *Dlx5* knockdown cells by retrovirus-mediated ectopic expression of active Notch3 (NIC3) or activated Akt2 (MyrAkt2). (**E**) immunoblot analysis depicting re-expression of active forms of Notch3 and Akt2 in F86-793 line (Note: MSCV-mediated transduction delivers high expression of genes encoding Notch3 or constitutively activated Akt2, which required only very short film exposure times; thus, the relatively much weaker endogenous Notch3 or p-Akt are not discernable here). (**F**) decreased growth of *Dlx5* knockdown cells is partially rescued by ectopic expression of NIC3 or activated Akt2. Cells were inoculated subcutaneously into NSG mice, and tumor weights were measured after 2 weeks. Significance: *p < 0.05; **p < 0.01; ***p < 0.005; ****p < 0.001; *****p < 0.0005.

To determine if Dlx5 is still required after tumor initiation, knockdown of *Dlx5* in *Lck-Dlx5* tumor cells was achieved using retroviral-mediated expression of shRNA. *Dlx5* knockdown resulted in reduced tumor cell viability/proliferation (Figure 3A) and an increased population of apoptotic sub-G1 cells (Figure 3B; Supplementary Figure 3A) due, in part, to down regulation of phospho-Akt and/or Notch1/3 (Figure 3C). To test whether overexpression of active Notch or Akt could reverse the knockdown effect, cells with knockdown of *Dlx5* were infected with retroviral *NIC3* or *MyrAkt2*; cell viability was measured 3 d after transduction and the knockdown effect was rescued (Figure 3D; Supplementary Figure 3B). We next inoculated cells into immunocompromised NSG mice via subcutaneous injection, and tumor weight was assessed 2 weeks after injection. We found that Dlx5 expression was required for tumor growth *in vivo* and that knockdown of *Dlx5* could be partially rescued by expression of either NIC3 or *MyrAkt2* (Figure 3E, 3F).

### Activation of both Notch and Akt are essential for survival and proliferation of *Lck-Dlx5* lymphoma cells

**Figure 4:**
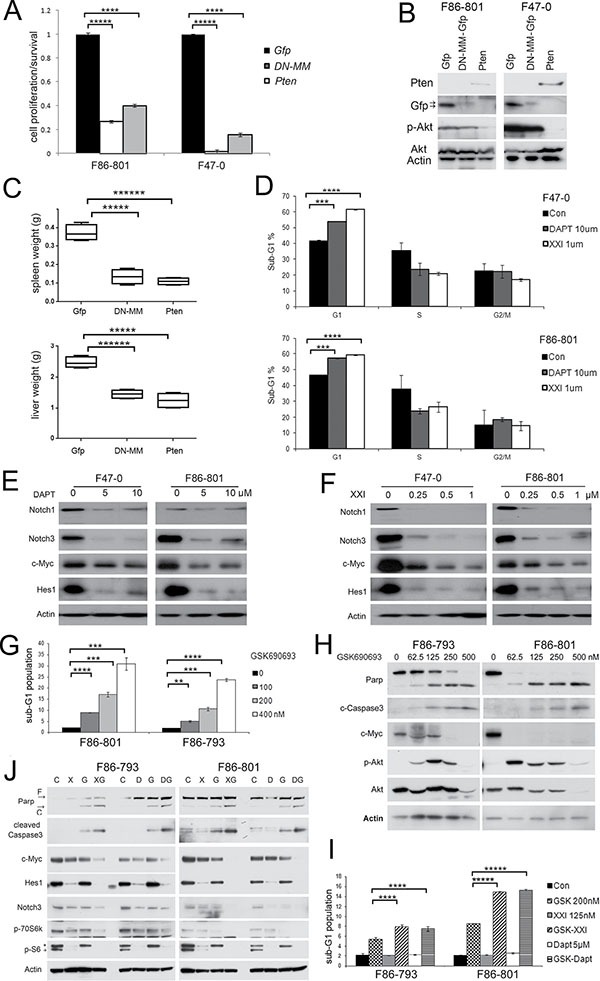
Akt and Notch signaling are essential for proliferation and survival of *Lck-Dlx5* lymphoma cells (**A**) ectopic expression of *Pten* or dominant-negative *mastermind* (*DNMM*) inhibits survival of *Lck-Dlx5* tumor cells as shown by MTS assay. Control was ectopically expressed *Gfp*. (**B**) immunoblot analysis depicting expression of exogenous *DNMM*-*Gfp* and *Pten*. (**C**) tumor growth of tail vein-injected *Lck-Dlx5* lymphoma cells into NSG mice. Weights of tumor cell-infiltrated liver and spleen are shown. (**D**) flow cytometry showing that Notch inhibitors XXI or DAPT suppress cell cycle progression of *Lck-Dlx5* tumor cells. (**E** and **F**) Notch inhibitors decrease Notch1, Notch3, Hes1 and c-Myc protein levels. Cells were harvested 24 h after beginning treatment with inhibitor. (**G** and **H**) Akt inhibitor GSK690693 alone triggers sub-G1 DNA condensation, caspase 3 activation, and Parp cleavage in *Lck-Dlx5* tumor cells. Upregulation of phospho-Akt in GSK690693-treated cells is indicative of a feedback loop to Akt, although downstream effectors of Akt signaling, e.g., p-mTOR and p-p70S6k are downregulated [44]. (**I** and **J**) when cells were treated with GSK690693 plus Notch inhibitors XXI or DAPT, apoptosis was significantly increased, as assessed by flow cytometry and immunoblotting. *, residual Hes1 band due to previous blotting with Hes1 antibody. Significance: *p < 0.05; **p < 0.01; ***p < 0.005; ****p < 0.001; *****p < 0.0005.

To directly block the Notch pathway, we transduced GFP-coupled *Dominant negative mastermind* (*DN-MM*) into *Lck-Dlx5* lymphoma cells, which resulted in reduced cell viability. Similarly, inhibition of the Akt pathway by re-introducing Pten expression reduced cell proliferation (Figure 4A, 4B). We next injected *Lck-Dlx5* lymphoma cells into the tail vein of NSG mice. The *Lck-Dlx5* lymphoma cells formed nodules primarily in liver and spleen 2 weeks after intravenous injection (Supplementary Figure 4A). Spleen and liver weights were measured to estimate lymphoma growth in these two organs. Inhibition of Akt or Notch signaling in *Lck-Dlx5* lymphoma cells by transduction of Pten or *DN-MM*, respectively, markedly suppressed tumor growth *in vivo* (Figure 4C).

Previous studies have demonstrated that the majority of human T-ALL cell lines are resistant to γ-secretase inhibitors due largely to mutations of *PTEN* or *FBXW7* [20, 21]. Interestingly, although expression of Pten protein was absent in *Lck-Dlx5* lymphoma cells, these cells were still sensitive to Notch inhibitors. Treatment with DAPT or γ-secretase inhibitor XXI for 2 days strongly suppressed the proliferation of *Lck-Dlx5* lymphoma cells, as shown by cell cycle analysis (Figure 4D; Supplementary Figure 4B). Moreover, immunoblotting revealed that Notch signaling is diminished following treatment with γ-secretase inhibitors (Figure 4E, 4F). The mTOR inhibitor RAD001 enhanced the inhibitory role of Notch inhibitors, as demonstrated by a further reduction in cell viability (Supplementary Figure 4C) and cell cycle arrest (Supplementary Figure 4D, 4E). When combined with XXI or DAPT, RAD001 strongly inhibited expression of Cyclin A and Cyclin D1 (Supplementary Figure 4F). Additionally, the Akt inhibitor GSK690693 induced apoptotic sub-G1 DNA condensation within 20 h (Figure 4G; Supplementary Figure 4G), which was also accompanied by activation of caspase-3 (Figure 4H), consistent with Akt's pro-survival role being independent of mTOR. Moreover, combinational treatment of *Lck-Dlx5* tumor cells with GSK690693 and DAPT or γ-secretase inhibitor XXI resulted in a marked increase in cell death as shown by augmentation of the sub-G1 peak and by activation of caspase-3 (Figure 4I, 4J; Supplementary Figure 4H). Collectively, these findings indicate that activation of the Akt and Notch pathways contribute independently to Dlx5-mediated oncogenesis.

### *Notch1* and *Notch3* are direct targets of Dlx5

**Figure 5:**
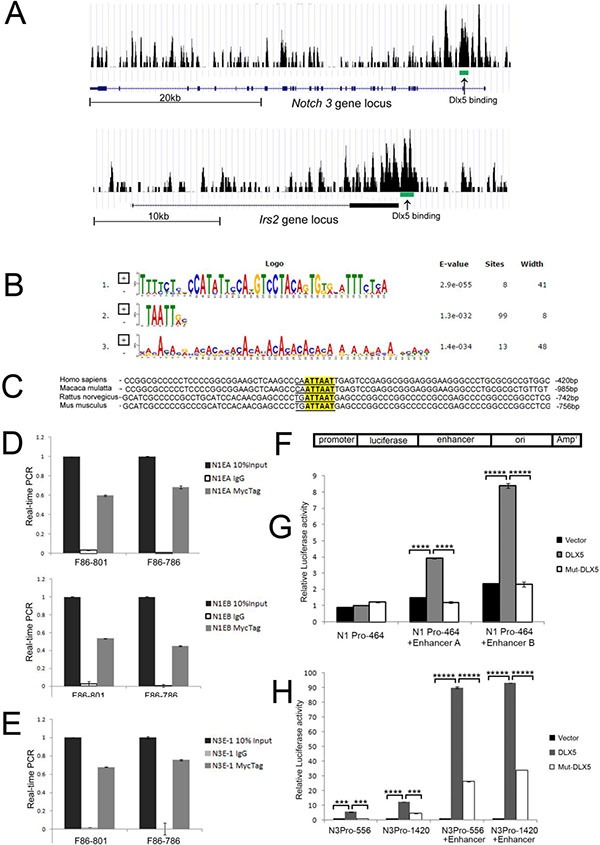
*Notch1* and *Notch3* are direct downstream targets of Dlx5 (**A**) ChIP-seq analysis revealed Dlx5 binding sequences in *Notch3* (MACS *p* value: −10*LOG10 = 70.54) and Irs2 (MACS *p* value: −10*LOG10 = 76.57) loci as depicted in screenshots from UCSC Genome Browser. Arrows point to binding intervals in each locus. (**B**) Multiple Em for Motif Elicitation (MEME) shows that the majority of the Dlx5 binding consensus site belongs to the classic binding motif (TAATT) of the Dlx family. (**C**) Irs2 promoters from human, monkey, rat and mouse contain Dlx5 binding consensus. (**D**) results of ChIP-qPCR assay confirmed that Dlx5 binds to two enhancer elements at end of *Notch1* gene. (**E**) ChIP-qPCR verified that Dlx5 binds to enhancer element located in *Notch3* intron 2. (**F**) schematic diagram of *Notch1* and *Notch3* promoter/enhancer reporter plasmids. Luciferase assay confirmed that Dlx5, but not Dlx5 mutant lacking the DNA binding domain, activates *Notch1* (**G**) and *Notch3* (**H**) promoters upon binding to their respective enhancer elements.

ChIP-seq was used to discover Dlx5 target genes. Dlx5 was found to bind to promoter or enhancer regions of various genes, including *Notch1, Notch3, Irs2 and Ccnd1* (Figure 5A, Supplementary Figure 5A). Multiple Em for Motif Elicitation (MEME) analysis revealed that the majority of Dlx5 consensus binding belongs to the classic binding motif (TAATT) of the Dlx family, which served as an internal quality control for our ChIP-Seq experiment (Figure 5B). BLAST analysis demonstrated that the Dlx5 binding consensus on the *Irs2* promoters is conserved among human, monkey, rat and mouse (Figure 5C). ChIP-qPCR confirmed that Dlx5 specifically binds to *Notch1* and *Notch3* enhancer elements (Figure 5D, 5E). The mouse *Notch1* and *Notch3* promoter fragments were sequentially cloned into pGL3 vector. Two enhancer elements downstream of the *Notch1* gene were inserted separately into pGL3 downstream of the *Notch1* core promoter sequence (N1P1) at a *Sal*I site within the vector (Figure 5F). In addition, the enhancer element located in intron 2 of *Notch3*, downstream of a 600-bp promoter fragment containing the core promoter (N3P1), was also cloned into pGL3. HEK293T cells were then co-transfected with pGL3-*Notch1* promoter/enhancers or *Notch3* promoter/enhancer and WT or mutant Dlx5 plasmids. Luciferase activity was measured 16 h after transfection. The reporter assay demonstrated that wild-type Dlx5, but not a Dlx5 mutant missing the DNA binding domain, augments transcription of the *Notch1* promoter in the presence of *Notch1* enhancer A or B (Figure 5G). Likewise, Dlx5 expression enhanced the luciferase activity of the *Notch3* promoter when its enhancer was inserted into the same plasmid (Figure 5H). Interestingly, other members of the Dlx family, i.e., Dlx4 and Dlx6, also were capable of upregulating Notch1 and Notch3 luciferase activity via their enhancers (Supplementary Figure 5B, 5C).

### Dlx5 induces early alteration of Notch and Akt signaling during T-cell development

**Figure 6:**
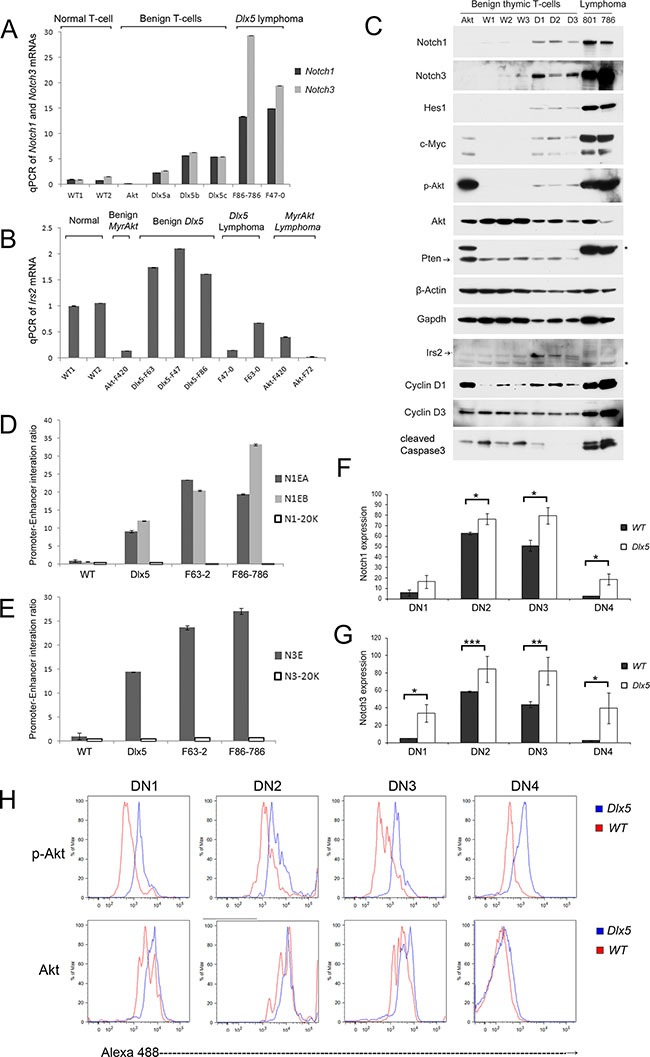
Dlx5 induces activation of Notch and Akt during T-cell development Real-time PCR was performed on thymic T cells from 5-week-old *Lck-Dlx5* mice (D1, D2, D3) prior to tumor development, WT littermates (W1,W2,W3), a 5-week-old *Lck-MyrAkt2* mouse (Akt), and *Lck-Dlx5* lymphomas (801, 793) to compare expression levels of full-length *Notch1/3* (**A**) and *Irs2* (**B**). (**C**) immunoblotting confirming increased expression of Notch1/3 in the non-malignant T cells from *Lck-Dlx5* mice, with further enhanced expression in tumors from these mice. (**D** and **E**) CCC assay showing frequency of interaction of Dlx5-bound *Notch1* enhancers to *Notch1* promoter (D) and Dlx5-bound *Notch3* enhancer to *Notch3* promoter (E). N1-20K and N3-20K refer to control DNA segments 20 Kb downstream of *Notch1* or *Notch3* loci, respectively. (**F** and **G**) Flow cytometry analysis of various stages of T-cell development showing elevated expression of Notch1 (F) and Notch3 (G) in all DN stages (DN1-DN4) in *Lck-Dlx5* mice compared to that of WT mice. (**H**) Flow cytometry analysis revealing increased expression of activated Akt kinase (p-Akt) in all DN stages in T cells from *Lck-Dlx5* mice.

Real-time PCR showed that in 5-week-old mice, *Notch1* and *Notch3* mRNAs are upregulated in normal thymocytes from *Lck-Dlx5* mice when compared to those of normal thymic T cells from age-matched WT littermates or *Lck-MyrAkt2* mice, and their expression was further augmented in lymphomas from *Lck-Dlx5* mice (Figure 6A). In contrast, *Notch2* and *Notch4* mRNAs were not altered in the tested samples (Supplementary Figure 6A). *Hes1* and *Myc*, two target genes of Notch signaling, also showed elevated mRNA levels (Supplementary Figure 6B). Additionally, transcript levels of *Irs2* and *Ccnd1*, two genes identified by ChIP-seq analysis as Dlx5 targets, also were upregulated (Figure 6B; Supplementary Figure 6B). Immunoblotting confirmed that normal thymic T cells from *Lck-Dlx5* mice have elevated Notch1/3 protein expression compared to that of normal thymic T cells from WT mice. Further upregulation of Notch1/3 protein levels was observed in *Lck-Dlx5* lymphoma cells (Figure 6C). The reason why mRNA levels of *Notch1* and *Notch3* were 2–4 fold greater in lymphoma cells than in normal T cells from *Lck-Dlx5* mice was not due to gene amplification, as array-CGH analysis revealed no change of *Notch1/3* gene copy number. Instead, the increased gene transcript levels in tumor cells appeared to result from increased interaction between *Notch* promoters and Dlx5-bound *Notch* enhancers, as revealed by CCC assay (Figure 6D, 6E). Furthermore, flow cytometry analysis revealed that Notch1/3 protein levels are elevated early on during T-cell development from CD4-CD8- Double-Negative (DN1 [CD25- CD44+]; DN2[CD25+ CD44+]; DN3 [CD25+ CD44-]; and DN4 [CD25- CD44-]) stages (Figure 6F, 6G; Supplementary Figure 6C). Importantly, the increased full-length Notch protein levels resulted in elevated Notch cleavage (Supplementary Figure 6D). Hes1 and Myc proteins, targets of active Notch, were also upregulated in normal T cells from *Lck-Dlx5* mice (Figure 6C; Supplementary Figure 6E, 6F). This finding suggests that upregulation of Notch1/3 might serve as the first cooperating oncogenic “hit” in T-cell lymphomagenesis in *Lck-Dlx5* mice.

Interestingly, phospho-Akt levels were also consistently elevated in normal T cells from *Lck-Dlx5* mice (Figure 6C). This increase was found to occur at the early stage of T-cell development from DN1 to DN4, CD4/CD8 DP and CD4-positive cells, but not in CD8-positive cells (Figure 6H; Supplementary Figure 6G). We previously reported that in human ovarian carcinoma cells, DLX5 transactivates the *IRS2* gene via direct binding to the *IRS2* promoter, thereby resulting in enhanced AKT signaling. Similar findings were documented in T cells from *Lck-Dlx5* mice, based on ChIP-seq analysis (Figure 5A). We found that Irs2 transcript and protein levels are increased in non-malignant T cells from *Lck-Dlx5* mice, but not in their malignant T-cell counterparts that have lost expression of Pten protein (Figure 6B, 6C). Loss of Pten expression results in constitutive activation of Akt, circumventing the need for upregulation of Irs2. Thus, it appears that Irs2-related Akt activation is a driving force from the very beginning of Dlx5-provoked T-cell lymphomagenesis, with more profound activation due to Pten loss occurring in fully malignant cells.

### Dlx5 promotes T cell survival during b-selection via Notch and Akt

**Figure 7:**
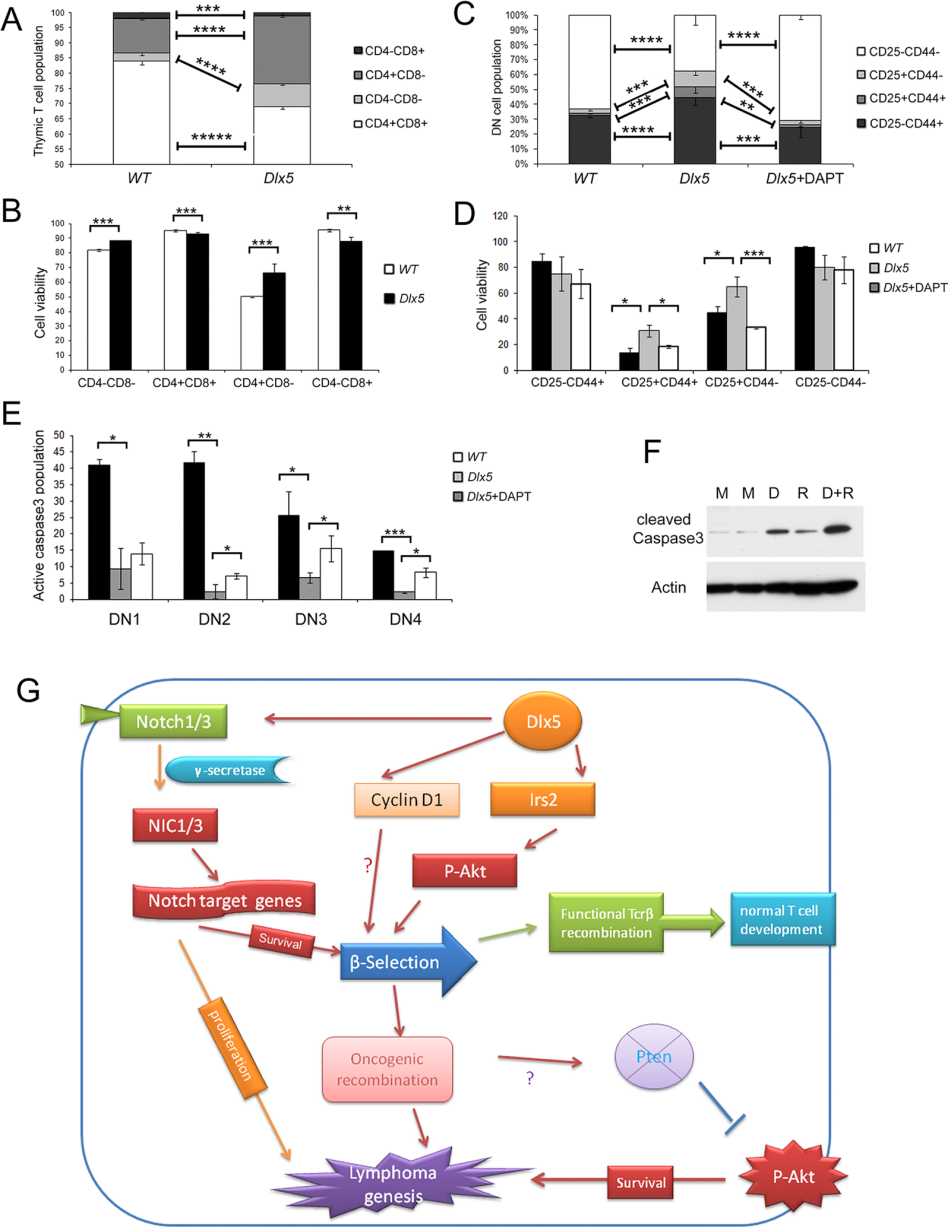
Dlx5 conveys survival signals via Notch and Akt during β-selection (**A**) Flow cytometry analysis of thymic T cells from 5-week-old WT and *Lck-Dlx5* mice demonstrating that *Lck-Dlx5* mice have increased percentage of CD4-CD8- cells and decreased percentage of CD4+CD8+ cells. (**B**) modest but statistically significantly increased survival of CD4-CD8- (DN) T cells from *Lck-Dlx5* mice, as shown by Annexin V/PI staining. (**C**) CD25 and CD44 staining showing increased population of CD25+CD44- cells in thymus of Lck*-Dlx5* mice. DN1 = CD25− CD44+; DN2 = CD25+ CD44+; DN3 = CD25+CD44−; and DN4 = CD25- CD44−. Administration of DAPT for 5 d resulted in a decrease in this cell population. (**D**) annexin V/PI staining demonstrating that CD25+CD44− population in *Lck-Dlx5* mice has increased cell viability compared to that of DN3 cells from WT mice, and that DAPT treatment reverses Dlx5′s pro-survival effect. (**E**) staining for cleaved caspase3 showing that T cells from *Lck-Dlx5* mice have much less caspase3 activation at the DN3 stage compared to that of T cells from WT mice, which was partially rescued by treatment with DAPT. (**F**) immunoblot analysis demonstrating that treatment of T cells from *Lck-Dlx5* mice with DAPT (D) and/or RAD001 (R) results in increased gross cleavage of caspase3. M = mock (placebo) treatment. (**G**) proposed mechanism of Dlx5-induced lymphomagenesis. Aberrant expression of Dlx5 in immature thymic T cells results in direct binding to *Notch1* and *Notch3* enhancer sequences as well as to promoters of *Irs2* and *Ccnd1*, which activates expression of these critical target genes. Resulting augmented Notch and Akt signaling promotes survival of affected immature T cells during β-selection. Fully transformed T cells continue to rely on Notch and Akt signaling for proliferation and dissemination via subsequent mutation of *Notch1* and loss of Pten.

Immunoblot analysis revealed that non-malignant thymic T cells from *Lck-Dlx5* mice have significantly less activation of caspase-3 than tumor cells (Figure 6C; Supplementary Figure 7A). Flow cytometry analysis showed that *Lck-Dlx5* mice have less CD4+CD8+ DP cells, with more cells at the DN stage (Figure 7A). This is likely due to the fact that the DN population in *Lck-Dlx5* mice has increased viability, consistent with reduced AnnexinV/PI staining (Figure 7B; Supplementary Figure 7B). Moreover, the DN population in *Lck-Dlx5* mice had less caspase-3 activation (Supplementary Figure 7C, 7D). CD44 and CD25 staining demonstrated that *Lck-Dlx5* mice have an increased T cell population at the CD44-CD25+ DN3 stage (Figure 7C), and AnnexinV/PI staining indicated augmented cell survival at this stage (Figure 7D). Furthermore, DAPT treatment for 5 d reversed the enhanced viability of the DN3 cell population in *Lck-Dlx5* mice (Figure 7D; Supplementary Figure 7E), consistent with caspase-3 activation in DN3 cells (Figure 7E; Supplementary Figure 7F). Nevertheless, treatment of *Lck-Dlx5* mice with DAPT or RAD001 resulted in increased gross cleavage of caspase-3 in thymic T cells (Figure 7F). The mechanism of Dlx5-induced T-cell lymphomagenesis is summarized in Figure 7G.

## DISCUSSION

Cancer may be considered as development gone awry. Multiple homeobox genes have been implicated in tumor formation. Chromosomal rearrangements with breakpoints in TCR genes are common in T-cell malignancies. These rearrangements place TCR regulatory sequences beside T-cell-related transcription factor genes, leading to their oncogenic activation. For example, one recurrent rearrangement, inversion of chromosome 7, inv(7)(p15q34), is observed in a subset of patients with T-ALL or T-NHL [3, 22]. This rearrangement repositions the *HOXA* gene on chromosome band 7p15 adjacent the *TRB* locus at 7q34, which is analogous to the inversion inv(6)(A2B1) we reported in T-cell lymphomas from *Lck-MyrAkt2* mice [6].

Little is known about the target genes of homeoproteins, although transfection studies with HOXA10 revealed upregulation of several genes encoding components of the Wnt pathway, including *WNT10B* and *FZD1*, which are essential in hematopoietic stem cell renewal [23]. Notably, Soulier and colleagues determined that the HOXA-related subgroup of T-ALL is characterized by high levels of expression of several genes known to be critical for T-cell differentiation and/or oncogenesis, including *NOTCH3* [3], which we found to be transcriptionally regulated by Dlx5 in lymphomas from *Lck-Dlx5* mice. Interestingly, two of the four *HOXA10*-rearranged cases also had prototypical *NOTCH1* mutations, suggesting that *HOXA10* and *NOTCH1* abnormalities cooperate oncogenically in such T-ALLs. Notably, 60% of tumors from our *Lck-Dlx5* mice also harbored activating mutations of *Notch1*. During hematopoietic stem cell differentiation, Notch1 drives the transition of lymphoid progenitor cells toward T lymphocytes [24], and *Notch1* is considered a master regulator of T-cell fate specification [25]. Moreover, Notch signaling promotes survival of pre-T cells at the b-selection checkpoint by regulating cell metabolism [26]. In human T-ALL, *NOTCH1* is targeted by activating mutations in 50–70% of cases [14, 27]. Collectively, these clinical data, like our experimental findings in *Lck-Dlx5* mice, are consistent with a model in which developmental homeobox genes play a central role in T-ALL oncogenesis.

Pear et al. demonstrated that intracellular *Notch1* (*ICN1*) could directly drive murine T-cell lymphoma formation [28]. However, most gain-of-function *NOTCH1* mutations found in human T-ALL do not generate downstream signals of sufficient strength to efficiently initiate leukemia development in mice [29]. Nevertheless, such weak gain-of-function *NOTCH1* alleles were able to hasten the onset of T-cell malignancy initiated by other leukemogenic events, such as activation of K-ras. Unlike *NOTCH1*, *NOTCH3* mutations have not been reported in human T-ALL, although NOTCH3 is often highly expressed [30]. It is also notable that although Dlx5 was expressed in the two *Lck-MyrAkt2* lymphoma cell lines shown in Figure 2C, Notch1 and Notch3 levels were not upregulated. Moreover, as shown in Figure 2C, among three *Lck-MyrAkt2* cell lines that expressed Dlx5 (lines 19–21), real-time PCR analysis revealed transcriptional upregulation of *Notch1/3* in only one (line 21). One possible reason for this is that a Notch1/3 enhancer-specific coactivator generally may be lacking in *Lck-MyrAkt2* lymphoma cell lines expressing Dlx5. In any case, the collective findings indicate that *Lck-Dlx5*-driven lymphomagenesis differs from that of *Lck-MyrAkt2*-driven thymic lymphoma formation. Specifically, in *Lck-MyrAkt2* mice, constitutively active Akt2 alone may serve as such a potent oncogenic factor that Myc activation, either directly via a *Myc* translocation or indirectly through its transcriptional activation by Dlx5, is sufficient as a “second hit.” In contrast, in *Lck-Dlx5* mice, which have a longer tumor latency than *Lck-MyrAkt2* mice, multiple oncogenic factors, including Notch1/3, Irs2, Cyclin D, and Myc, appear to be required to initiate tumor formation.

Recently, *NOTCH1* was identified as a direct target of DLX5 during squamous cell differentiation [31]. Using combinatorial unbiased approaches entailing ChIP-seq and microarray analysis, we determined that Dlx5 binds to both the *Notch1* and *Notch3* loci and promotes their transcription. *Lck*-*Dlx5* transgenic mice show upregulation of Notch1 and Notch3 throughout T-cell differentiation, and basal levels of Notch1/3 were further augmented in malignant T-cells due to increased enhancer-promoter interaction. Moreover, thymic T cells from *Lck-Dlx5* mice were resistant to apoptosis during b-selection at the DN3 stage, and such resistance could be reversed by treatment with γ-secretase inhibitors. Thus, we concluded that Dlx5 directly transactivates *Notch1*/*3* genes and promotes β-selection escape, which eventually results in tumor formation (Figure 7G). While lymphoma cells from *Lck-Dlx5* mice were sensitive to γ-secretase inhibitors, those from *Lck-MyrAkt2* mice (most of which did not show over expression of Notch1/3; Figure 2) were resistant, further strengthening our contention that Notch signaling is a driving force in Dlx5-mediated lymphomagenesis. Our study of human pediatric T-ALL revealed overexpression of DLX5 in about 20% cases, with co-upregulation of *NOTCH1* and, to a lesser extent, *NOTCH3*. Intriguingly, not all homeobox genes behave in a similar manner, as a recent report showed that TLX1 actually inhibits *NOTCH3* expression [32].

During T-cell development, IL-7 induces a much greater increase in Pi3k-Akt activity via Irs2 than by Irs1, suggesting an important role for Irs2 in T-cell differentiation [33]. Moreover, Irs2-deficient T cells show defects in IL-4-induced proliferation and differentiation in mice [34]. Recent studies demonstrated that ~10% of T-ALL specimens display activating *IL7R* mutations resulting constitutive activation of JAK/STAT5 and PI3K/Akt/mTOR pathways [35, 36]. Interestingly, our ChIP-seq studies revealed that *Irs2* is a direct target of Dlx5, and *Lck-Dlx5* mice exhibit elevated Akt signaling beginning early in T-cell differentiation via enhanced expression of Irs2, which would be expected to augment Akt activity and facilitate Notch-induced lymphomagenesis.

AKT signaling is frequently activated (~50%) in human T-ALLs, mostly due to alterations of *PTEN*, *PI3K*, and *AKT* genes [37, 38]. We previously reported that DLX5 contributes to AKT signaling in human ovarian cancer cells via direct upregulation of *IRS2* transcription [11]. When overexpressed, IRS2 has been shown to cause mammary tumorigenesis and metastasis [39]. In this investigation, we found that all lymphomas from *Lck*-*Dlx5* mice have loss of Pten protein expression due to mutation/deletion or epigenetic silencing [13], resulting in constitutive activation of Akt. Thus, in both *Lck-MyrAkt2* mice and *Lck-Dlx5* mice, tumor formation appears to require cooperation between Akt and Dlx5 or Myc. In *Lck-MyrAkt2* mice, we proposed that activation of the Akt pathway acts as the initial “hit” to promote cell survival and genomic instability, whereas the acquisition of T-cell-specific overexpression of Dlx5 leads to lymphomagenesis [40]. In *Lck-Dlx5* mice, on the other hand, Akt activation due to Pten loss may act as a second hit during Dlx5-driven T cell lymphomagenesis.

In summary, our studies show that Dlx5 initiates T-cell lymphomagenesis via direct binding to *Notch1/3* and *Irs2* genes to augment their transcription, which switches on Notch and Akt signaling, respectively, during T-cell fate decisions. The resulting spontaneous T-cell lymphomas maintain addiction to Dlx5 through further intensified Notch and Akt signaling, thus connecting the Dlx5 homeoprotein to two pathways that are also known to be critically involved in the proliferation and survival, respectively, of human T-ALL. More generally, these experimental findings provide mechanistic insights about how transcriptional reactivation of a homeobox gene can drive T-ALL development via epigenetic reprogramming.

## MATERIALS AND METHODS

### Primary tumors and tumor-derived cell lines

*Lck-Dlx5* mice were examined daily and sacrificed upon evidence of difficulty in breathing, severe weight loss, or when tumor burden is otherwise obvious, under IACUC approval in accordance with NIH guidelines. At the time of death due to lymphoma development, a portion of each tumor was snap-frozen in liquid nitrogen, another portion was kept in Trizol for Microarray, and the remaining portion was placed into culture in an attempt to establish tumor cell lines for subsequent Chip-Seq, immunoblotting, and MTS assays as well as for *in vitro* studies with pathway inhibitors. For microarray expression analysis and real-time PCR, primary tumor samples were used.

### Reagents

Antibodies against Dlx5, Notch3, cyclin A, cyclin D1, Irs2, Gapdh and β-actin were from Santa Cruz Biotechnology; antibodies against Notch1, Notch1 (Val1744), Pten, p-Akt, Akt, Myc-Tag, Myc-Tag-Alexa647, and cleaved caspase-3-Alexa488 were from Cell Signaling. Akt pathway inhibitors GSK690693 and RAD001 were from Selleckchem (Houston, TX). Hes1 luciferase vector and Notch3 plasmid were from AddGene. MSCV-MyrAkt2 plasmid was as described [11], and MSCV-GFP-DN-MM plasmid was kindly provided by Warren S. Pear (UPenn) [41].

### Cell viability assay

Cell viability was assessed by MTS assay (Promega). Lymphoma cell lines from *Lck-Dlx5* mice were seeded at 1 × 10^4^ cells/well in 96-well plates and treated with inhibitors for 24 or 48 h. OD value at 490 nm was measured 2–4 h after incubation of cells with 10% MTS using a 96-well microplate reader (BioRad).

### Western blot analysis

Protein from lymphoma cell lines from *Lck-Dlx5* mice was extracted with cell lysis buffer supplemented with 2 mM PMSF (Cell Signaling); 50 μg/well proteins were loaded into Tris-Glycine SDS-PAGE gels (Invitrogen) and transferred onto PVDF membranes (Millipore). Protein blots were incubated with antibodies overnight at 4°C. Signals were developed after incubation with HRP-conjugated secondary antibody.

### Microarray analysis

Primary lymphoma samples from several different founder lines were subjected to mRNA microarray analysis using Affymetrix GeneChip Mouse Gene 2.0 ST Arrays (Affymetrix). Primary tumor mRNAs from *Lck-Dlx5* mice (F86-801, F47-918, F63-0) and *Lck-MyrAkt2* (F72-3148, 3153, 3237) mice, as well as mRNA from thymic T cells of wild-type (WT) mice, were extracted and subjected to microarray hybridization. CEL files were analyzed with Nexus expression 3.0 software (BioDiscovery, El Segundo, CA) to discover the differentially expressed genes and associated pathways. Data set has been submitted to GEO (# GSE83685).

### ChIP-seq analysis

ChIP-seq was performed and analyzed on Lck-Dlx5 cell line F85-786 by Active Motif. Myc-Tag antibody, as well as IgG control, were used to immunoprecipitate Dlx5/chromatin complexes, and captured DNA fragments were analyzed by Illumina GA II to obtain whole-genome datasets (available at GEO #GSE83778). MACS p-Value was used to make all peak callings.

### Dlx5 knockdown by retroviral-mediated short-hairpin RNA (shRNA) interference

Sequences used for *Dlx5* knockdown were selected using shRNA Design Guidelines (Ambion Technical Bulletin #506, Life Technologies). shRNAs were synthesized, annealed and inserted into pSilencer (Invitrogen). Two constructs with best knockdown efficiency were used for experiments. Viruses were produced as described [42]. In brief, 293T cells were co-transfected with retroviral vector EcoPac and plasmid. Mouse lymphoma cell lines were infected at a MOI of 1.5 for 6 h.

### Promoter and enhancer cloning

Promoter regions and enhancer elements of mouse *Notch1* and *Notch3* genes were cloned by PCR using Q5 DNA polymerase (New England Biolabs). Promoter fragments were cloned into pGL3 basic vector (Promega), and enhancer elements were inserted in the *Sal*I site of promoter vector pGL3-core. Plasmids were transfected into 293T cells, and luciferase activity was measured using a Dual-Luciferase reporter assay kit (Promega) on an Enspire alpha plate reader (Perkin Elmer).

### Chromatin immunoprecipitation (ChIP) assay

ChIP assays were performed using EZ-ChIP (Millipore) per manufacturer's instructions. Lymphoma cell lines from *Lck-Dlx5* mice were fixed using 1% formaldehyde, stopped by adding glycine to a final concentration of 125 mM. DNA:protein complexes were then resuspended in 0.1% SDS lysis buffer and sonicated into 200-1000-bp fragments. Chromatin was immunoprecipitated overnight at 4°C with Myc-tag antibody (Abcam). Immunocomplexes were eluted with 1%SDS, 0.1 M NaHCO_3_, de-crosslinked with 5 M NaCl, and incubated at 65°C overnight. PCRs of the target loci were performed using specific primers and Power SYBR Green Master Mix (Thermo Fisher Scientific).

### Chromosome conformation capture (CCC)

CCC methodology and primers were as described [43]. Lymphoma cell lines from *Lck-Dlx5* mice were fixed with 2% formaldehyde for 5 min, neutralized with glycine, and lysed with buffer containing 0.2% NP-40, 10 nM Tris HCl (pH 8.0), and 10 mM NaCl for 12 h. DNA was digested with *Eco*RI or *Bam*HI overnight, followed by ligation with T4 DNA ligase and extraction of DNA with phenol. Interaction frequencies between promoter and enhancer were analyzed by real-time PCR.

### Flow cytometry

Flow cytometry was performed with a LSRII machine (Becton Dickinson) to analyze T-cell developmental and cell death markers from healthy Lck-Dlx5 mice. CD4-APC/Cy7, CD8-PE, CD44-APC/Cy7, CD25-PE, Notch1-Alexa647 and Notch3-APC antibodies were obtained from BioLegend. Annexin V-FITC and ethidium homodimer III were from Biotium. Data were analyzed with FlowJo software.

## SUPPLEMENTARY MATERIALS FIGURES AND TABLES




