# Supplementary material for: The homeoprotein Dlx5 drives murine T-cell lymphomagenesis by directly transactivating Notch and upregulating Akt signaling
Source: Oncotarget. 2017 Jan 21;8(9):14941–56. doi: 10.18632/oncotarget.14784 (PMC5362456; doi:10.18632/oncotarget.14784)
Supplement: Supplementary file 1 [file oncotarget-08-14941-s001.pdf]

The homeoprotein Dlx5 drives murine T-cell lymphomagenesis by directly transactivating notch and upregulating Akt signaling

Supplementary Materials

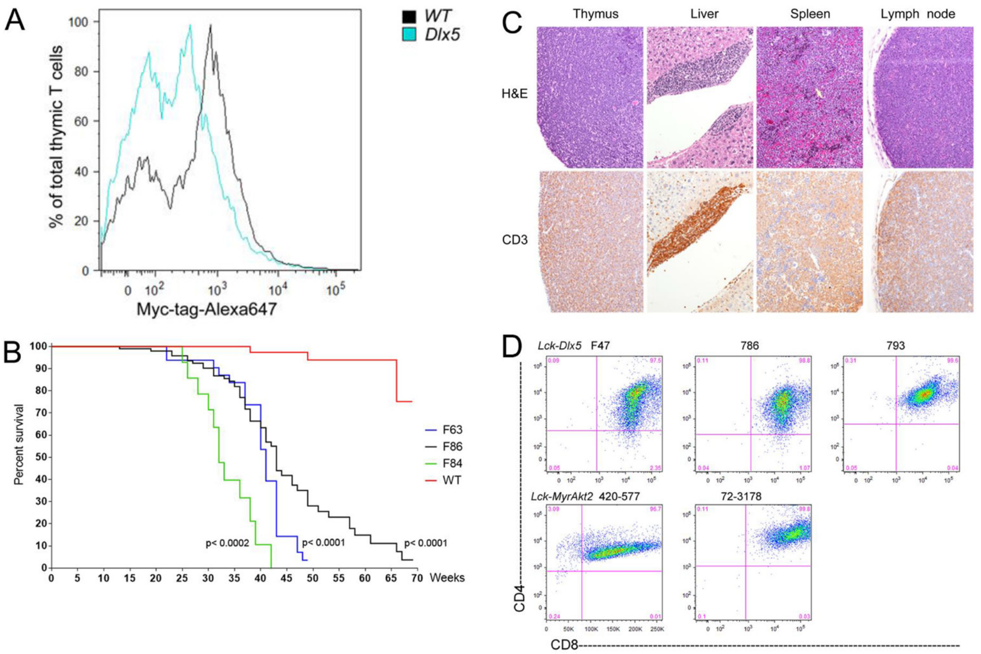

Supplementary Figure 1: Pathological characterization of T-cell lymphomas from Lck-Dlx*5* mice. (**A**) Flow cytometry demonstrating that thymic T cells from 5-week-old *Lck-Dlx5* mice express abundant levels of Dlx5 protein. T cells were harvested by passing thymus tissue through a 100-µM mesh, fixed, permeabilized and stained with Alexa647-labeled Myc-Tag antibody. (**B**) Survival curves from three founder lines of *Lck-Dlx5* transgenic mice. (**C**) *Lck-Dlx5* lymphomas are of T cell origin. FFPE tissue sections were stained with antibody against the T cell surface marker CD3. The primary lymphoma in thymus as well as the invasive lymphoma in liver, spleen and lymph node show CD3 positive staining. (**D**) Flow cytometric analysis indicating that *Lck-Dlx5* lymphoma cells are CD4+CD8+ double positive. Three T-cell lymphoma lines derived from *Lck-Dlx5* mice, as well as two T-cell lymphoma lines from *Lck-MyrAkt2* mice (also CD4+CD8+), were stained with CD4-FITC and CD8-PE and analyzed by flow cytometry.

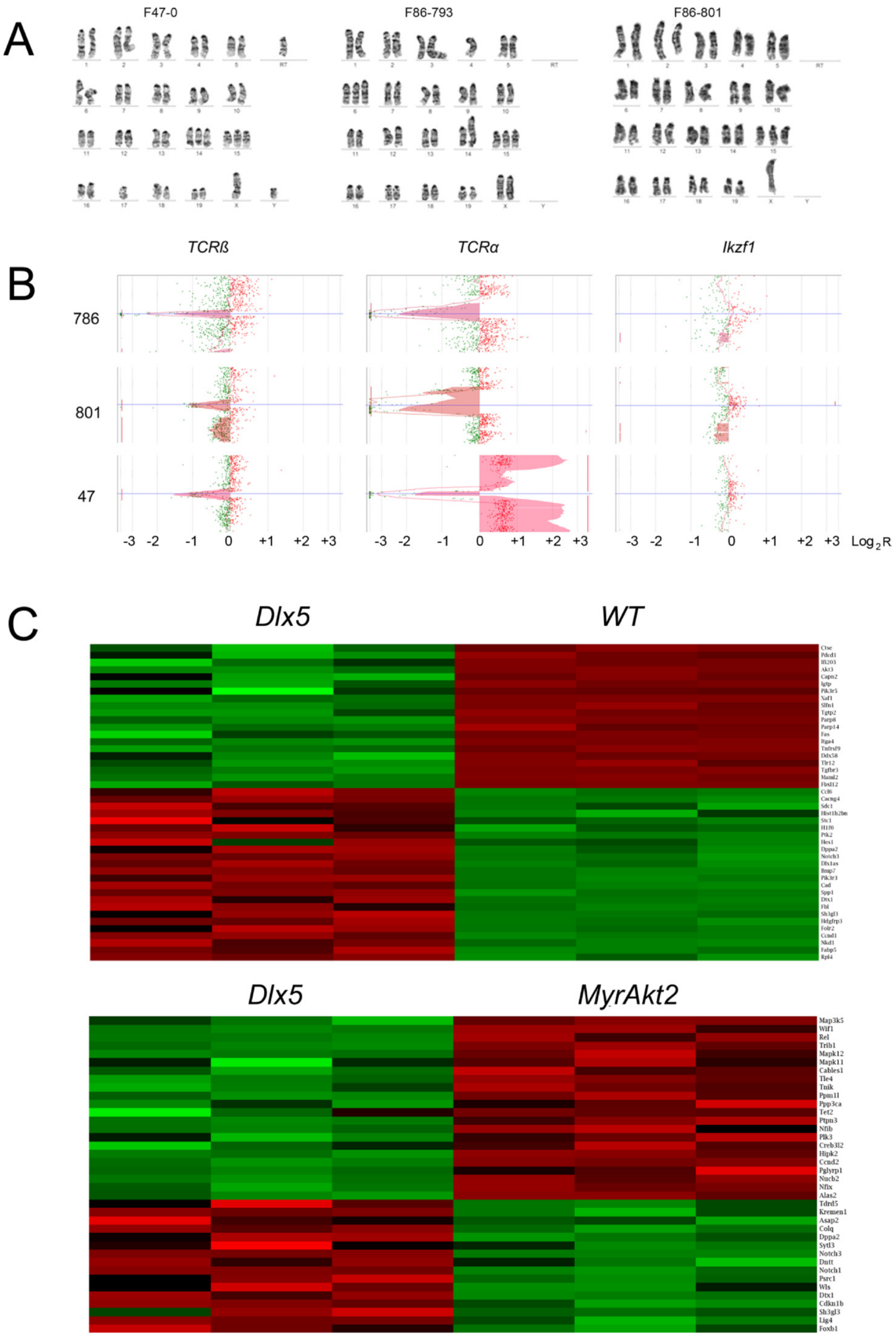
A
F47-0
F86-793
F86-801
B
TCRß
TCRα
Ikzf1
786
801
47
-3 -2 -1 0 +1 +2 +3
Log2R
C
Dlx5
WT
Ctse
Pdcd1
Ifi203
Akt3
Capn2
Igtp
Pik3r5
Xaf1
Slfn1
Tgtp2
Parp8
Parp14
Fas
Itga4
Tnfrsf9
Ddx58
Tlr12
Tgfbr3
Maml2
Fbxl12
Ccl6
Cacng4
Sdc1
Hist1h2bn
Stc1
H1f0
Ptk2
Hes1
Dppa2
Notch3
Dlx1as
Bmp7
Pik3r3
Cad
Spp1
Dtx1
Fbl
Sh3gl3
Hdgfrp3
Folr2
Ccnd1
Nkd1
Fabp5
Rpl4
Dlx5
MyrAkt2
Map3k5
Wif1
Rel
Trib1
Mapk12
Mapk11
Cables1
Tle4
Tnik
Ppm1l
Ppp3ca
Tet2
Ptpn3
Nfib
Plk3
Creb3l2
Hipk2
Ccnd2
Pglyrp1
Nucb2
Nfix
Alas2
Tdrd5
Kremen1
Asap2
Colq
Dppa2
Sytl3
Notch3
Dntt
Notch1
Psrc1
Wls
Dtx1
Cdkn1b
Sh3gl3
Lig4
Foxb1

D

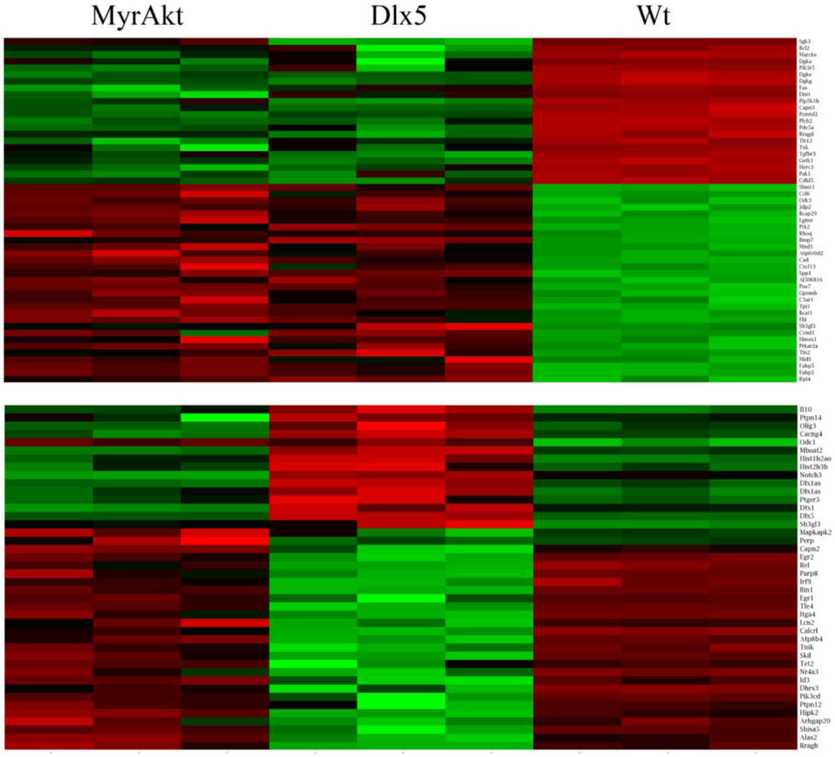

MyrAkt
Dlx5
Wt
Sgk3
Bcl2
Marcks
Dgka
Pik3r5
Dgke
Dgkg
Fas
Dntt
Pip5k1b
Capn3
Pcmtd2
Plcb2
Pde5a
Rragd
Tlr12
Txk
Tgfbr3
Gstk1
Herc3
Pak1
Cdkl5
Shmt1
Ccl6
Odc1
Jdp2
Bcap29
Lgmn
Ptk2
Rhoq
Bmp7
Mnd1
Atp6v0d2
Cad
Cxcl13
Spp1
AI506816
Pus7
Gpnmb
C3ar1
Tpi1
Bcat1
Fbl
Sh3gl3
Ccnd1
Hmox1
Prkar2a
Tln2
Mid1
Fabp5
Fabp5
Rpl4
Il10
Ptpn14
Olig3
Cacng4
Odc1
Mboat2
Hist1h2ao
Hist2h3b
Notch3
Dlx1as
Dlx1as
Ptger3
Dtx1
Dlx5
Sh3gl3
Mapkapk2
Perp
Capn2
Egr2
Rel
Parp8
Irf9
Bin1
Egr1
Tle4
Itga4
Lcn2
Calcrl
Atp8b4
Tnik
Skil
Tet2
Nr4a3
Id3
Dhrs3
Pik3cd
Ptpn12
Hipk2
Arhgap20
Shisa5
Alas2
Rragb

E

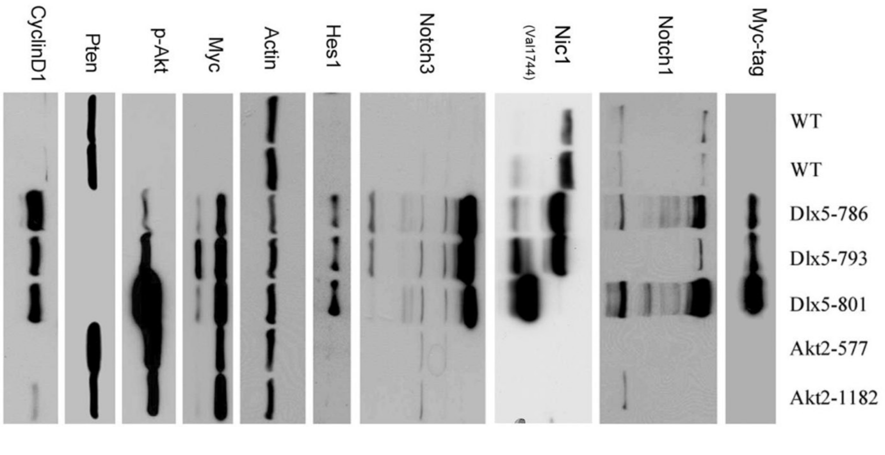

CyclinD1
Pten
p-Akt
Myc
Actin
Hes1
Notch3
(Val1744)
Nic1
Notch1
Myc-tag
WT
WT
Dlx5-786
Dlx5-793
Dlx5-801
Akt2-577
Akt2-1182

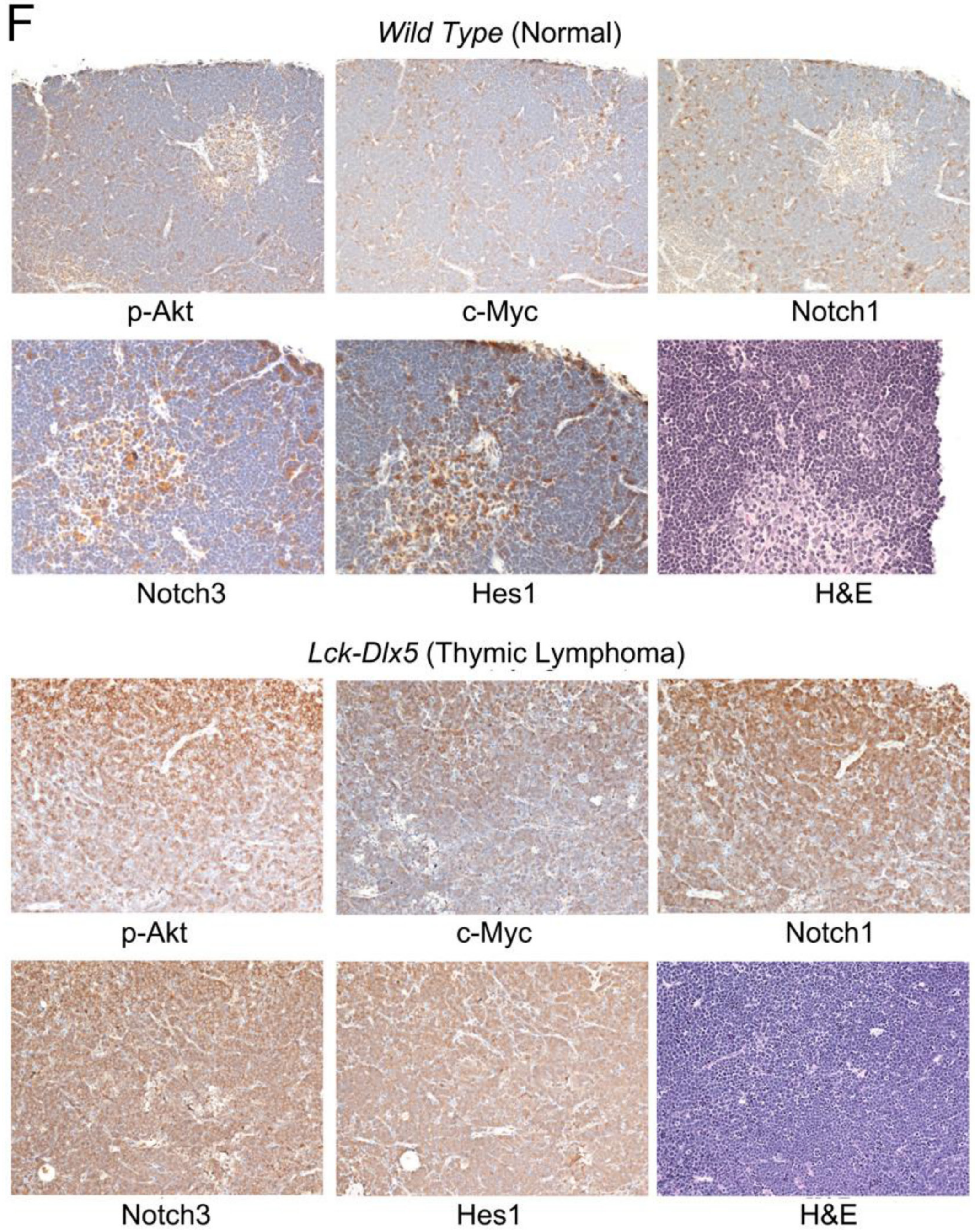

Supplementary Figure 2: Genomic features and expression profile of T-cell lymphomas from *Lck-Dlx5* mice. (**A**) Trisomy 15 present in three representative thymic lymphoma T-cell lines from mice of two different *Lck-Dlx5* founder lines. In addition to variable numerical and structural chromosome abnormalities, trisomy 15 is present in each cell line shown. (**B**) Array-CGH analysis reveals that thymic lymphoma cell lines from *Lck-Dlx5* mice are of monoclonal origin. Green dots mean loss, red dots mean gain. Peaks represent loss or gain with statistical significance. Data were analyzed by using Genomic Workbench from Agilent. Note unique genomic patterns at loci for TCRα (*Tcra*), TCRß (*Tcrb*) and Ikaros zing finger (*Ikzf1*). (**C**) Expression heatmaps demonstrated upregulated genes in *Lck-Dlx5* lymphomas compared with those observed in normal T cells from wild-type (WT) mice (upper panel), as well as downregulated genes in *Lck-Dlx5* lymphomas (lower panel). Red = up regulated genes; green = down regulated genes (upper panel). (**D**) Expression heatmaps demonstrated commonly expressed genes in *Lck-Dlx5* lymphomas and *Lck-MyrAkt2* lymphomas compared with those observed in normal T cells from wild-type (WT) mice (upper panel). Uniquely expressed genes in *Lck-Dlx5* lymphoma cells compared with those of normal T cells from WT mice and T-cell lymphoma cells from *Lck-MyrAkt2* mice (lower panel). (**E**) Immunoblot analysis demonstrating unique pattern of oncogenic alterations in tumors from Lck-Dlx5 mice versus that of tumors from *Lck-MyrAkt2* mice and T cells of WT mice. (**F**) H&E and immunohistochemical staining of T-cell lymphoma tissues from *Lck-Dlx5* mice and normal thymus from WT mice. Note strong staining for Notch1, Notch3, Hes1, Myc and phospho-Akt in primary *Lck-Dlx5* thymic tumors.

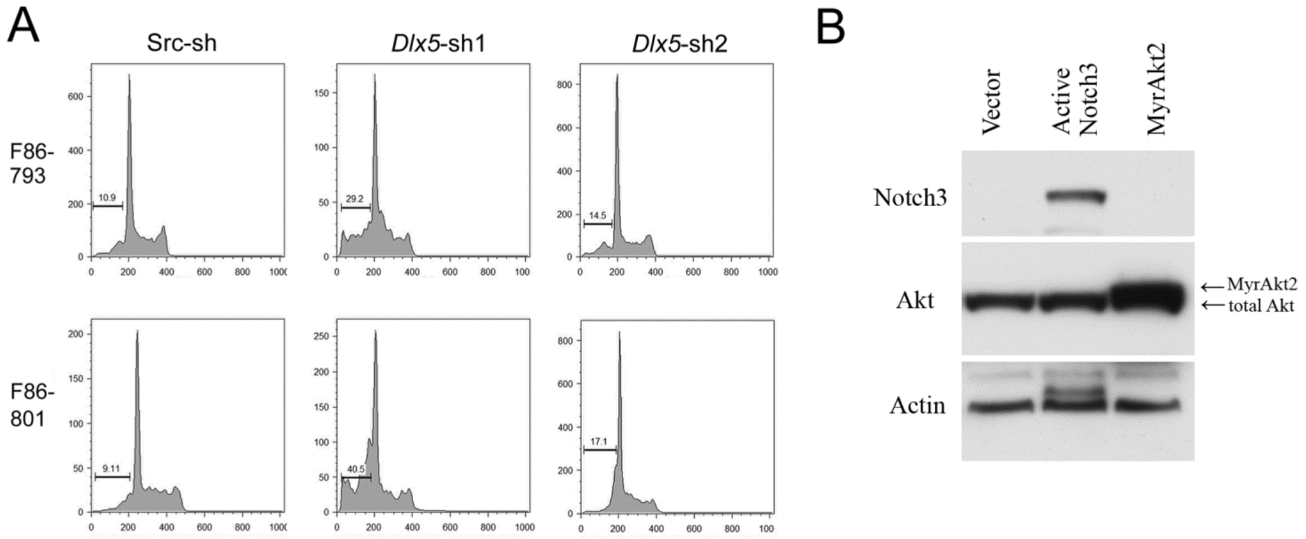

Supplementary Figure 3: Dlx5 is required for the survival of *Lck-Dlx5* lymphoma cells. (**A**) *Dlx5* lymphoma cells were transduced by retrovirus-mediated *Dlx5* shRNAs *in vitro*. A representative set of flow cytometry charts shows apoptotic sub-G1 population. (**B**) *Dlx5* knockdown can be rescued by activated Notch or Akt. *Lck-Dlx5* lymphoma cell line F86-801 with shRNA-mediated knockdown of *Dlx5* was retrovirally-transduced with *MyrAkt2* or active *Notch3* (NIC3). Vec, empty vector; Scr. scrambled shRNA control.

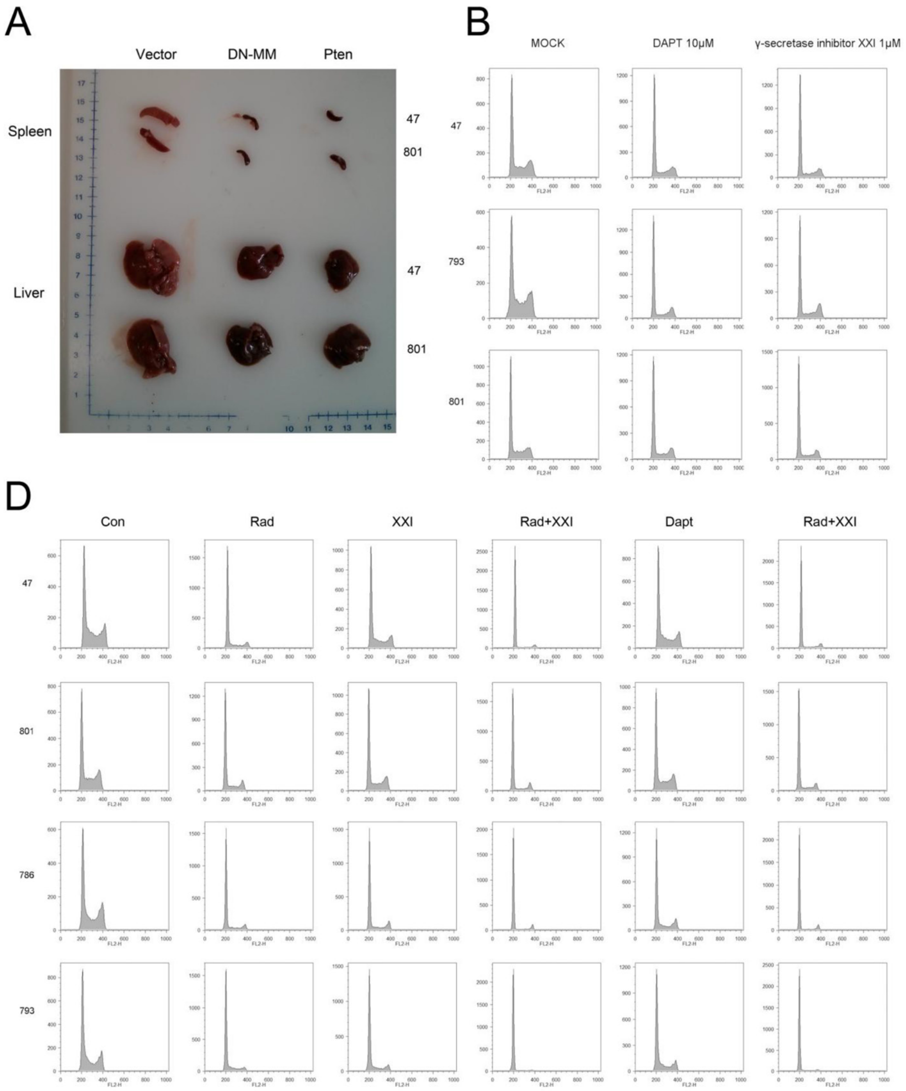

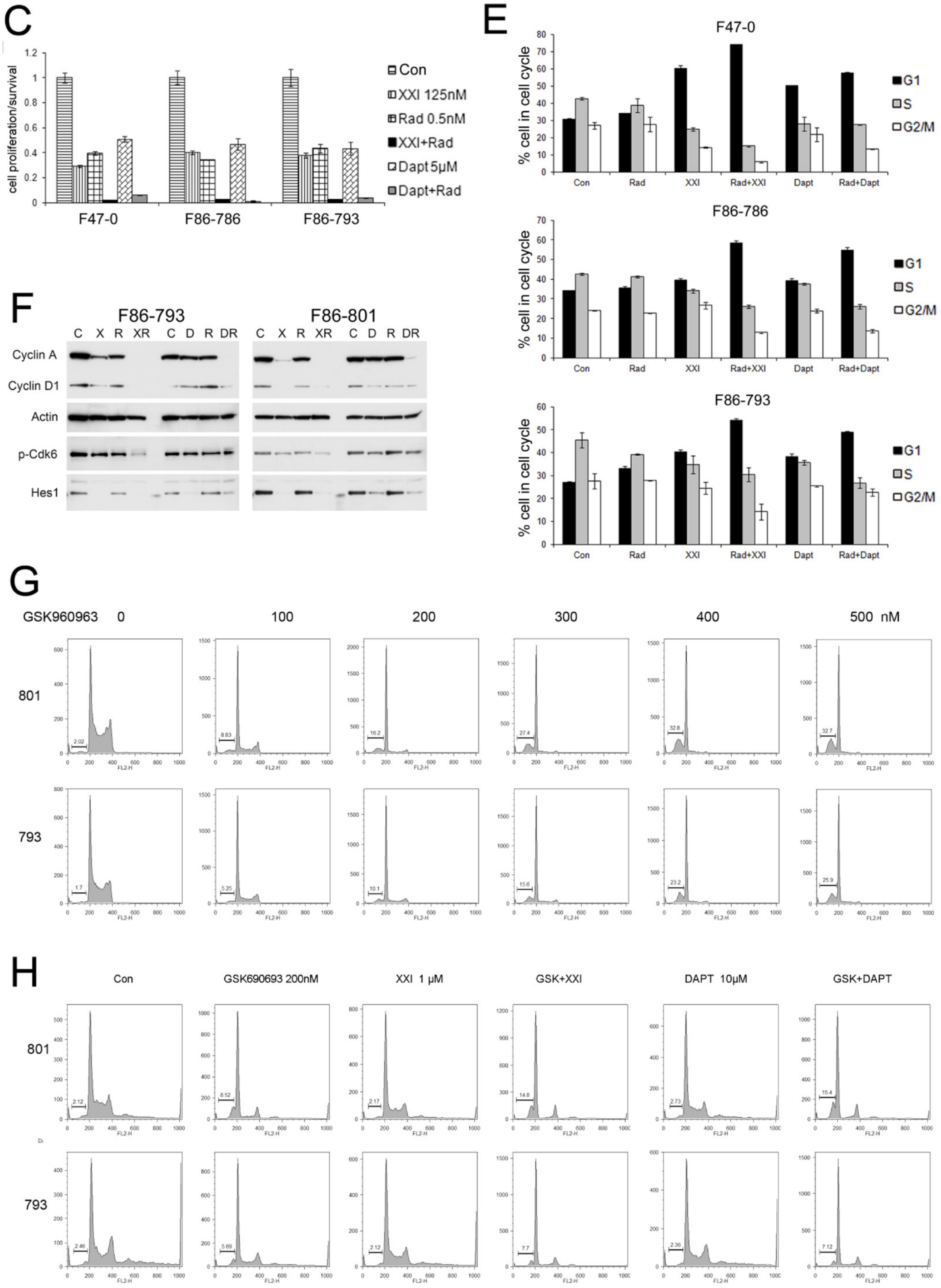

Supplementary Figure 4: Cooperative signaling by activated Akt and Notch are essential to proliferation and survival of *Lck-Dlx5* lymphoma cells. (**A**) Inhibition of Notch or Akt signaling suppresses the growth of *Dlx5* tumors *in vivo*. *Lck-Dlx5* lymphoma cells were transduced with the inhibitory *DN-MM* or *Pten*, inhibitors of Notch and Akt signaling, respectively. Stable cell lines were injected into NSG mice via tail vein, and liver and spleen were weighted and photographed after 2 weeks. (**B**) DAPT and γ-secretase inhibitor XXI trigger G1/S cell cycle arrest in *Lck-Dlx5* lymphoma cells. Logarithmically proliferating lymphoma cells were fixed with ice-cold 70% EtOH, stained with PI and analyzed by FACS. (**C**) MTS assay demonstrating that RAD001 markedly enhances effect of Notch inhibition on cell viability (**D**–**E**) RAD001 enhances the effect of XXI and DAPT. Results for three different *Lck-Dlx5* lymphoma cell lines are shown. (**F**) Cyclin A and cyclin D1 were greatly downregulated in *Lck-Dlx5* cells treated with RAD001 (R) together with XXI (X) or DAPT (D). (**G**) Akt inhibitor GSK690693 triggers apoptosis in *Lck-Dlx5* lymphoma cells. (**H**) Notch inhibition enhances the apoptotic effect of GSK690693.

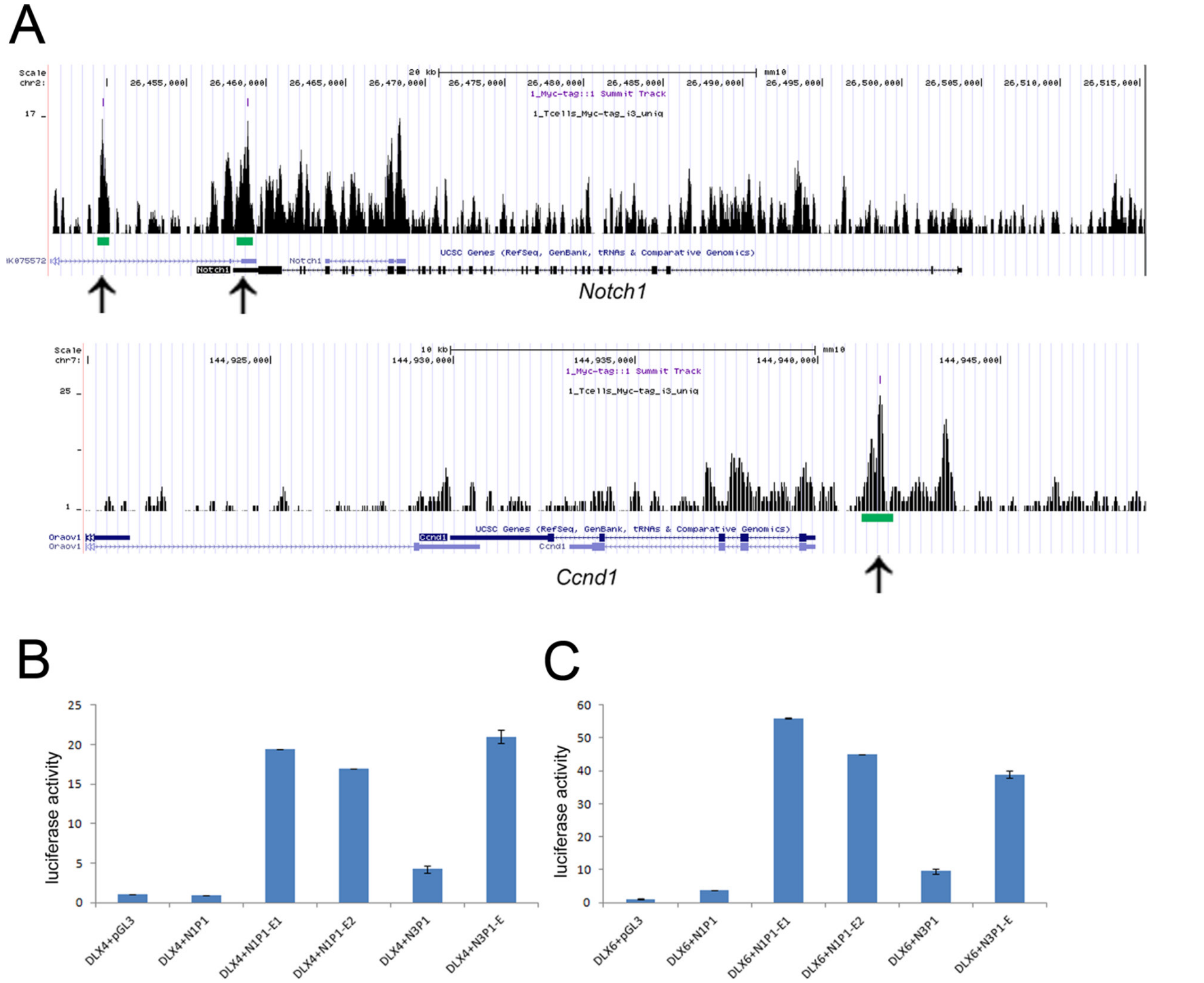

Supplementary Figure 5: *Notch1* and *Notch3* genes are direct downstream targets of Dlx family members. (**A**) ChIP-seq was used to discover Dlx5 target genes. Dlx5 was found to bind to promoter or enhancer regions of *Notch1* (MACS *p*-value -10*LOG10 = 118.69 for downstream enhancer and MACS p-value -10*LOG10=80.56 for in gene enhancer) and *Ccnd* (MACS *p*-value -10*LOG10 = 146.73) *1*. Like DLX5, DLX4 (**B**) and DLX6 (**C**) are capable of transactivating Notch1 and Notch3 via the identified enhancers as shown by luciferase reporter assay. N1P1 = Notch1 promoter 1; E1 and E2 = Notch1 enhancers; N3P1, Notch 3 promoter; E = Notch3 enhancer.

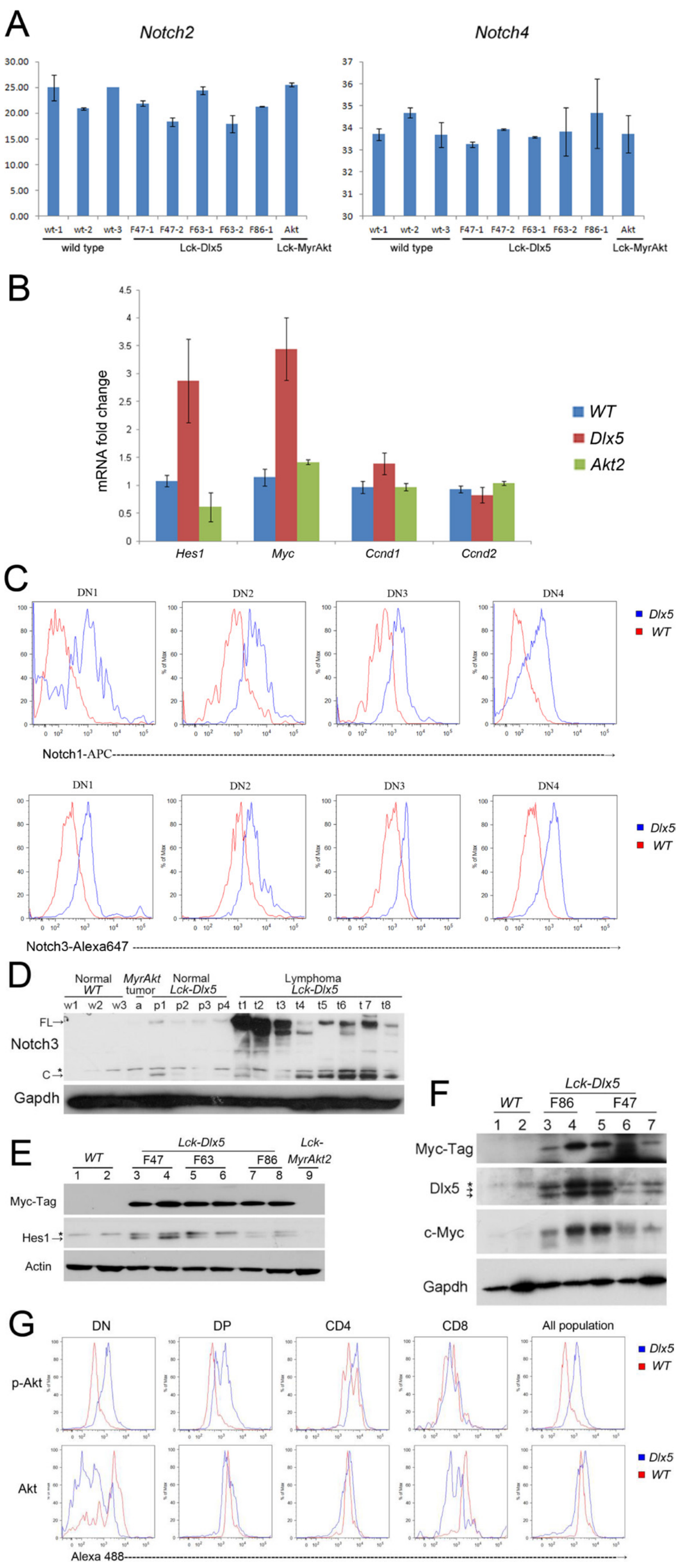

Supplementary Figure 6: Dlx5 induces early alteration of Notch and Akt signaling during T-cell development. (**A**) qPCR analysis of mRNA levels of *Notch2* and *Notch4* in benign (normal) thymic T cells from *Lck-Dlx5* mice and WT littermates. Note that unlike *Notch1* and *Notch3*, mRNA expression levels of *Notch2* and *Notch4* were not augmented in lymphomas from *Lck-Dlx5* mice. (**B**) qPCR analysis of mRNA levels of *Hes1*, *Myc*, *Ccnd1* and *Ccnd2* in normal T cells from *Lck-Dlx5* mice and WT littermates. (**C**) DN T-cells from *Lck-Dlx5* mice have elevated Notch1 and Notch3 protein levels. Normal thymic T cells from WT and *Lck-Dlx5* mice were stained with CD44, CD25 and Notch1-APC / Notch3-Alexa647antibodies, and were analyzed by flow cytometry. Immunoblotting (**D**, **E**, **F**) of normal thymic T cells from *Lck-Dlx5* mice show increased expression of Notch3 in full length and truncated forms (D), Hes1 (E) and Myc (F) proteins. Asterisks in panels D, E and F represent non-specific bands. (**G**) Thymic T-cells from *Lck-Dlx5* mice have elevated Akt activation. Normal thymic T cells from WT mice and Lck-Dlx5 transgenic mice were stained with CD4-Cy7APC, CD8-PE and p-Akt /Akt antibodies, then with Alexa488 labeled secondary antibody, and were analyzed by flow cytometry.

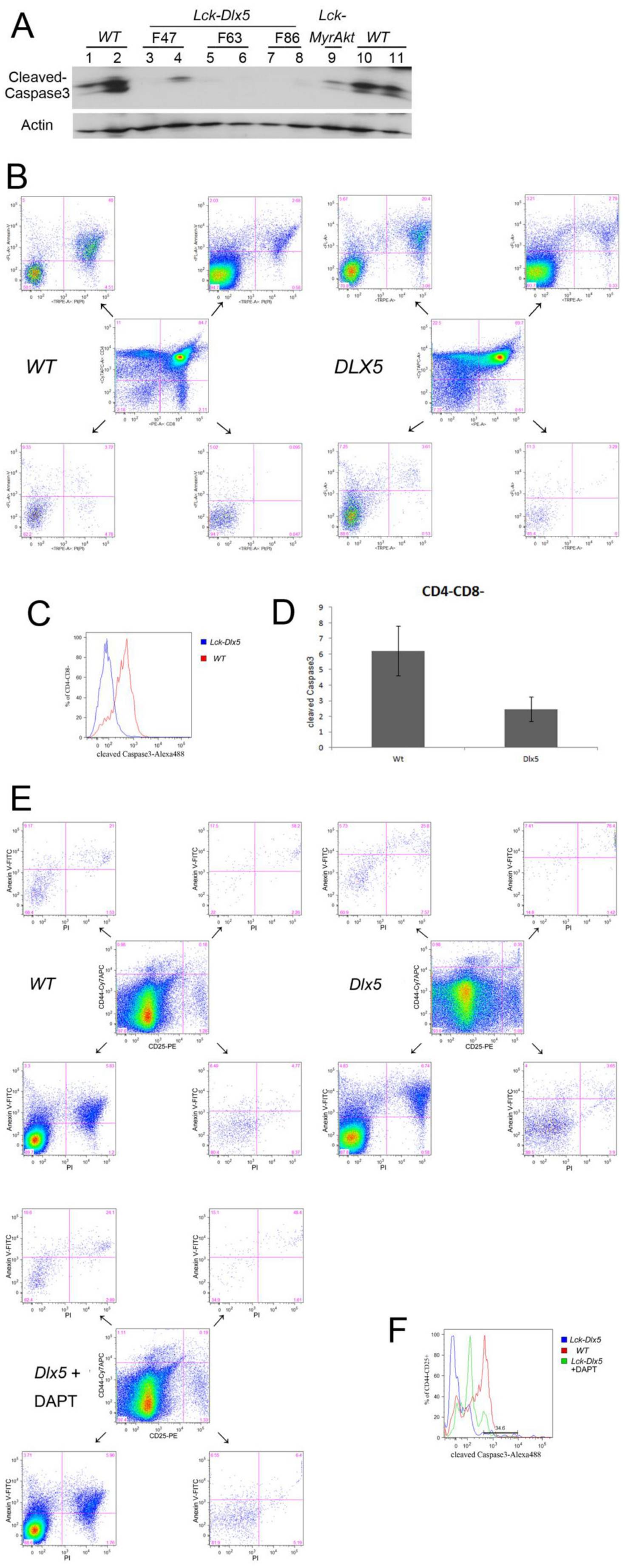
A
Lck-Dlx5
Lck-
MyrAkt
WT
F47
F63
F86
WT
1 2 3 4 5 6 7 8 9 10 11
Cleaved-
Caspase3
Actin
B
WT
DLX5
C
Lck-Dlx5
WT
% of CD4-CD8-
cleaved Caspase3-Alexa488
D
CD4-CD8-
cleaved Caspase3
Wt
Dlx5
E
WT
Dlx5
Dlx5 +
DAPT
Anexin V-FITC
PI
CD44-Cy7APC
CD25-PE
F
Lck-Dlx5
WT
Lck-Dlx5
+DAPT
% of CD44-CD25+
cleaved Caspase3-Alexa488
34.6

Supplementary Figure 7: Dlx5 enhances cell survival signaling via Notch during ß-selection. (**A**) Thymic T cells from 5-week-old mice were harvested. Immunoblotting shows that normal T cells from *Lck-Dlx5* mice and *Lck-MyrAkt2* mice have less gross cleavage of Caspase3 compared to that of WT mice. (**B**) Representative FACS analysis of thymic T cells from *Lck-Dlx5* mice, which exhibit less apoptosis than those of WT mice. Normal thymic T cells were stained with CD4/CD8 antibody in combination with Annexin V-FITC/PI. In T cells from *Lck-Dlx5* mice, there is an increased CD4/CD8 DN population when compared to that of WT mice (7.22% vs. 2.18%, respectively, in this representative flow cytometric analysis); additionally, the cell survival percentage among DN thymocytes is modestly increased in *Lck-Dlx5* mice vs. in WT mice (88.6% vs. 82.2%, respectively). (**C**, **D**) Flow cytometry of thymic T cells stained with CD4/CD8 in (**E**) Normal T cells were stained with CD44/CD25 in combination with Annexin V-FITC and PI. In the DN3 population (CD44− CD25+ cells), thymocytes from *Lck-Dlx5* mice show increased cell survival compared to that of WT mice (88.5% vs. 80.4%, respectively), which was reversed by the administration of DAPT *in vivo* (81.9%). (**F**) T cells were stained with CD44/CD25 in combination with cleaved Caspase3-Alexa488 antibody. In CD44−CD25+ DN3 population, *Lck-Dlx5* cells have less Caspase3 cleavage, which was partially reversed by *in vivo* injection of DAPT.

Supplementary Table 1: Karyotypic alterations identified in six cell lines derived from T-cell lymphomas of *Lck-Dlx5* mice

Cell line	Karyotypic alterations
86–7	+15, t(2;6)(G2;E2)
86–801	+15, t(2;6) (T2;B3)
86–786	+15
86–793	+15, dup(3)(E2;H4), +ins(6), ins(14)
47–0	+15, +14, −17
47–918	+15, +11

Supplementary Table 2: Sanger sequencing analysis of *Notch1* in cell lines derived from T-cell lymphomas of *Lck-Dlx5* mice. See Supplementary_Table_2
