# Supplementary material for: The homeoprotein Dlx5 drives murine T-cell lymphomagenesis by directly transactivating Notch and upregulating Akt signaling
Source: Oncotarget. 2017 Jan 21;8(9):14941–56. doi: 10.18632/oncotarget.14784 (PMC5362456; doi:10.18632/oncotarget.14784)
Supplement: Supplementary file 2 [file oncotarget-08-14941-s002.docx]

**Supplementary Table 2. Sanger sequencing analysis of *Notch1* in cell lines derived from T-cell lymphomas of *Lck-Dlx5* mice.**

| Cell line | Mutation |  | | Predicted consequence | |  |
| --- | --- | --- | --- | --- | --- | --- |
| F86-875 | CDS 7268 del CAGCAGTC^A^ |  | | nonsense mutation, truncated protein | | |
| F86-801 | CDS 6982-6990 insertion TATACTA^A^ | | | nonsense mutation, truncated protein | | |
| F86-786 | CDS 7081 ACG-ACA | |  | no change in aa sequence | |  |
| F86-793 | CDS 7083 ins A^A^ | |  | nonsense mutation, truncated protein | | |
| F86-1149 | CDS 5492 ACA-GCA^A^ | |  | missense Thr-Ala |  |  |
| F63-0 | CDS 7299 CGA-TGA^A^ | |  | nonsense mutation, truncated protein | | |
| F63-1263 | none | |  |  |  |  |
| F63-1210 | CDS 5002 CCT-CCC | |  | no change in aa sequence | |  |
| F47-0 | 5067 GGC-GCC^A^ | |  | missense Gly-Ala | |  |
| F47-1247 | none | |  |  |  |  |
| F47-918 | CDS 4720 CCT-CCC | |  | no change in aa sequence | |  |
| F84-1063 | CDS 7129 CAG-TAG^A^ | |  | nonsense mutation, truncated protein | | |
| F86-7 | CDS 7269-7276 deletion AGCAGTCT^A^ | |  | nonsense mutation, truncated protein | | |

^A^Activating mutation. Overall incidence of activating *Notch1* mutations was 61.5%.

**Oligos**

Cloning Myc-tagged full-length *Dlx5*

Forward: ATGACAGGAGTGTTTGACAGAAGA;

Reverse: CTACAGATCCTCTTCAGAGATGAGTTTCTGCTCCAAATAAAGCGTCCCGGAGG

Genotyping primers for *Lck-Dlx5* mice

For: ATGACAGGAGTGTTTGACAGAAGA;

Rev: ATGAAAAACGGGGAGATG

ShRNA sequence for Dlx5 knockdown

sh-1: GCGCAGCCAGCTCAATCAA

sh-2: TGAGGATGGTGAATGGTAA

sh-LacZ: GGATCAGT GCTGATTAAA.

*Notch1*

promoter cloning

F2536 AAACTTGACTTCAGCTGCTCC

F2609 TTCCTTTCCCACAAGACCC

F2201 TCGGACACAGCCAGGGAC

F2708 GCGCCAGCTCAAACTTTTGG

R3010 AGAGGCACTAGTGAGGCTCTG

R3160 TGATGCCCTCTTTCCTGGC

Notch1 enhancer A

cloning

F44498 GTATTGTTTTCTATGGCACTAGGG

R45526 CTAAGCCACAGTCTGACAGTCC

ChIP

| N1EA-F24768 | GCGTCACCTGGGATTTGCAGATG |
| --- | --- |
| N1EA-R24909 | CAAGTCCACACTGGTGGTTACAGAAAGTGTAC |
| N1EA-F25652 | GCAGTTGCCCACCTCCTCAGG |
| N1EA-R25752 | TCAACATCTTAGGATGCGTCTGGTCATATC |

Notch1 enhancer B

cloning

F53662 ACTGGAGCTGTGGACACTCTG

R54655 TCTGATAGCACCAGCATAGCAG

ChIP

| N1EB-F34080 | GCAGGGTATGCAACACCTGCACC |
| --- | --- |
| N1EB-R34201 | GAACGCTGCCAGCAGAGGGAG |
| N1EB-F34234 | TCCAGATGGCATTTGTGTCCATCCC |
| N1EB-F34342 | GAGTCATGTGGCTCTGGATCTAGTGAACC |

3C

| N1PromoterF4201ECORI30- | ATAGCCCCTTCCCGAGAACGG |
| --- | --- |
| N1PromoterF4201ECORI55 | TAAGATGGCGTGGGAGGGGC |
| N1Enhancer-AF12536ECORI55 | ATAGCTGGGTAGCTGCCTAGCCTCC |
| N1Enhancer-AF12536ECORI 35 | CTCCATATTGCTAAGACTCTGTCTCCCC |
| N1Enhancer-BR-14530ECORI30 | TTGCTAGTTTTAGTAGACTCACAGATTGTGGAATTC |
| N1Enhancer-BR-14530ECORI100 | CCAAGCAAAATGGGAACTTTCAGAAATT |
| N1Enhancer-BF-ECORI50 | TTCAACCCAATGCTCTCAGAATACTCAAC |
| N1Non-specificF-40KECORI48 | GAACTCGTCTCGAGGTGTTAACGTCC |
| N1Non-specificF-20K ECORI75 | CAACTAGGGCAGGTTCCCAGAAGC |
| N1Non-specificF-10K ECORI 55 | CAAGAGCTGAGGGGTCATTCAGTAGG |
| N1Non-specificF+40KECORI50 | CGCCAGCTGTGTCCTCTGTG |

*Notch3*

promoter cloning

F-2112 TCACCAACTCAGCCTAAGCC

F-1830 GGTGAGGACTGGAGATGTGG

F-1444 CCCTGGTCTCTGCATATGC

F-1042 TGTTTCAGTCCTTTGGTCTCC

F-580 AAGCACCTTTCCCTCTTCG

R+34 TCCCTCCTCCCTCTTTCC

enhancer cloning

F2681 CTGCTTGCCTGTGAGTGTCTG

R2823 GGGAAACACAAGAGGTTACCG

Enhancer ChIP

F2701 GTGAGTGTCTGGCCCAGCGC

R2800 AGCCACACACATGGAGGAAGTGATACC

Enhancer 3C

| N3PromoterF-1061BAMH135 | CAACTGACCTCAAACTAAGGCTGAGGTG |
| --- | --- |
| N3PromoterR-1131BAMH135 | CATCCCTTATAGCTTCTTTGTGGACCG |
| N3EnhancerF-7786BAMH170 | TAGGGTGACGCTAAGAAATGAGTCACC |
| N3EnhancerR-3871BAMH170 | GAAGCTGGAGATGAGCACGTGG |
| N3Non-specificF-20KBAMH160 | GTTGGGTGGGCTGCACAGTG |
| N3Non-specificF-10KBAMH160 | GTCCTGAGTTCAATTCCCAGCAACC |

Real-time PCR

| Notch1F352  Notch1R471  Notch3F340 | ACCTGCCTGAATGGAGGGAGGTA TTACACGGTGTGCTGAGGCAAGG GCCAGAGTTCAGTGGTGGCGG |
| --- | --- |
| Notch3R462 | AGGGGGCACCATGAACACAGG |
| Irs2f3891 | TGCCCTCTGCCAGCACCTATGC |
| Irs2r3986 | CGATGGGGCTGGTAGCGCTTC |
| Ctnnb1-F268 | CAAGTCCTTTATGAATGGGAGCAAGGC |
| Ctnnb1-R386 | AACATGGCAGCTCGGACCC |
| Ppib-F572 | ACCTCCTGGCTGGATGGCAAG |
| Ppib-R686 | ATCCTTCAGTGGCTTGTCCCGG |
